# Interactive effects of life cycle and monocot-dicot lineage on genome size–trait relationships in angiosperms: a phylogenetically informed analysis

**DOI:** 10.3389/fpls.2025.1647198

**Published:** 2025-08-29

**Authors:** Guolan Liu, Yin Wen, Peili Fu, Qiqi Cao, Qian Cui, Wen Du, Hao Chen, Wanli Zhao

**Affiliations:** ^1^ Shandong Key Laboratory of Eco-Environmental Science for Yellow River Delta, Shandong University of Aeronautics, Binzhou, Shandong, China; ^2^ Key Laboratory of Agro-Ecological Processes in Subtropical Region, Institute of Subtropical Agriculture, Chinese Academy of Sciences, Changsha, Hunan, China; ^3^ CAS Key Laboratory of Tropical Forest Ecology, Xishuangbanna Tropical Botanical Garden, Chinese Academy of Sciences, Jinghong, Yunnan, China; ^4^ Ailaoshan Station of Subtropical Forest Ecosystem Studies, Xishuangbanna Tropical Botanical Garden, Chinese Academy of Sciences, Jingdong, Yunnan, China

**Keywords:** angiosperms, functional traits, genome size (1C), monoploid genome size (1Cx), life cycle, monocot-dicot distinction

## Abstract

**Introduction:**

Genome size in angiosperms exhibits extraordinary variation, influencing a wide array of biological and ecological characteristics. Although prior studies have established links between genome size and certain functional traits, how the interaction between two fundamental axes of angiosperm diversity—life cycle (annual vs. perennial) and monocot-dicot distinction—shapes group-specific variation in genome size and its relationship with plant functional traits remains insufficiently understood.

**Methods:**

We assembled a comprehensive dataset encompassing 2,285 angiosperm species from 186 families, measuring genome size (1C-value) and monoploid genome size (1Cx-value) to account for polyploidy, as well as key size-related traits, including plant height and the length and width of leaves, flowers, fruits, and seeds. To evaluate the relationships between genome size and these functional traits while controlling for shared evolutionary history, we conducted phylogenetic generalized least squares (PGLS) analyses.

**Results:**

The results indicated that the interaction between life cycle and monocot-dicot distinction is a primary determinant of both 1C and 1Cx variation, with perennial monocots exhibiting the largest 1C and 1Cx values and a significant interactive effect between these two axes of diversity. Patterns of correlation between genome size metrics (1C, 1Cx) and functional traits are group-specific and sometimes reversed, reflecting divergent adaptive strategies—for example, genome size (1C) is positively correlated with plant height in annuals but negatively in perennials. Phylogenetic correction revealed that some associations, such as the negative correlation between genome size and plant height in perennials, are largely driven by shared ancestry and disappear after accounting for phylogeny, whereas others, such as the positive correlation between genome size (1C, 1Cx) and petal length, remain robust across groups, indicating a conserved adaptive relationship. The use of 1Cx confirmed that observed patterns reflect fundamental genome architecture rather than solely polyploidy effects.

**Discussion:**

These findings demonstrate that the interplay between life cycle and monocot-dicot distinction fundamentally shapes genome size (1C, 1Cx)–trait relationships in angiosperms, providing new insights into the evolutionary and adaptive mechanisms underlying plant diversity.

## Introduction

Genome size, measured as the haploid nuclear DNA content (1C value), exhibits over 2,100-fold variation among angiosperms (Pellicer et al., 2010; Bhadra et al., 2023). This remarkable diversity influences plant biology from cellular processes to whole-organism traits such as growth strategies, reproductive investment, and ecological interactions (Dodsworth et al., 2015; Sklenár et al., 2022; Van Mazijk et al., 2024). Recent studies have significantly advanced our understanding of genome size evolution, highlighting its role in plant adaptation to environmental gradients (Borokini et al., 2023; Guo et al., 2024a; Winterfeld et al., 2025), plant invasiveness (Guo et al., 2024b), and trait evolution during angiosperm radiation (Bhadra et al., 2023; Van Mazijk et al., 2024). However, the drivers and consequences of genome size diversity remain incompletely understood, particularly regarding the interplay between life cycle (annual vs. perennial), taxonomic identity (monocot vs. dicot), and plant functional traits.

Research has traditionally focused on simple pairwise relationships between genome size and cellular or organ-level traits, such as cell size, stomatal density, and seed mass (Beaulieu et al., 2008; Herben et al., 2012; Carta et al., 2022; Borokini et al., 2023). Recent advances reveal that these relationships are highly context-dependent, varying across clades, ecological settings, and evolutionary histories (Bhadra et al., 2023; Sklenár et al., 2022; Van Mazijk et al., 2024). For example, genome size can predict drought tolerance in Cape schoenoid sedges (Van Mazijk et al., 2024) and adaptive strategies in cosmopolitan grass species (Guo et al., 2024a), but such associations are not universal. Key axes of angiosperm diversity—life cycle and monocot-dicot distinction—reflect contrasting resource allocation strategies (Garnier and Laurent, 1994; Wiemann et al., 1998; Zarinkamar, 2006; Givnish et al., 2010; González-Paleo et al., 2019), yet their combined effects on genome size and associated traits have rarely been systematically tested.

The relationships between genome size and plant functional traits are far from universal; rather, they display remarkable variability across different clades, trait categories, and ecological contexts. Positive links (e.g., with seed weight in some taxa) coexist with negative associations (e.g., with maximum plant height; Knight et al., 2005) or non-significant relationships (Knight and Beaulieu, 2008). Such variability may arise from divergent life cycle strategies: annuals investing in rapid growth within a single season versus perennials developing persistent structures (Shao et al., 2021). Notably, species with the smallest genomes include both the shortest and tallest angiosperms (Shao et al., 2021), underscoring that trait relationships can reverse direction due to context-dependent selective pressures (Hessen et al., 2010; Hodgson et al., 2010; Malerba et al., 2020). This inconsistency highlights the complexity of genome size–trait linkages, suggesting that multiple, sometimes opposing, constraints may operate. A systematic approach is needed to clarify whether these patterns reflect adaptive divergence or phylogenetic legacy.

A major challenge in comparative plant biology is disentangling ecological or adaptive correlations from patterns imposed by shared ancestry. Closely related species often resemble each other not because of convergent evolution or adaptation, but simply due to inheritance from a common ancestor. To address this issue, phylogenetically informed analytical methods—such as phylogenetic generalized least squares (PGLS)—have become standard tools for testing trait correlations. For example, genome size–plant height relationships may stem from shared ancestry rather than adaptive convergence (Knight and Beaulieu, 2008). PGLS distinguish ancestral constraints from novel adaptations by correcting for evolutionary relatedness. However, applying these approaches to genome size-trait networks—while accounting for interactions between life cycle and monocot-dicot distinction—has been limited (Beaulieu et al., 2007; Shao et al., 2021).

While phylogenetic corrections are essential for addressing patterns of trait covariation across species, quantifying the tempo of genome size evolution through evolutionary rates (σ²) offers complementary insights into the processes shaping biological diversity (Ackerly, 2009). Rather than being limited to static trait correlations, evolutionary rate analyses enable researchers to investigate the temporal dynamics of trait evolution and to quantify the pace at which genome size diverges among lineages (Castiglione et al., 2018). Notably, some studies have suggested that evolutionary rates may be positively correlated with absolute genome size, such that larger genomes tend to evolve more rapidly than smaller ones (Malerba et al., 2020). Distinct evolutionary rates among functional groups may also reflect varying selective pressures or constraints linked to life history strategies and phylogenetic origins (Ackerly, 2009; Castiglione et al., 2018). For example, accelerated genome size evolution in certain clades could indicate episodes of adaptive radiation or periods of relaxed constraint, whereas slower rates may suggest the action of stabilizing selection or functional canalization (Malerba et al., 2020).

A further complication in uncovering true genome size–trait linkages arises from the prevalence of polyploidy in angiosperms. Polyploidy—whole-genome duplication—can dramatically increase total nuclear DNA content (1C-value) without necessarily altering the basic genome size per chromosome set (Bennett and Leitch, 2011; Suda et al., 2015; Guignard et al., 2016). This inflation in DNA content can confound analyses, as polyploid species often exhibit larger organs and cells, potentially exaggerating correlations between genome size and morphological traits (Beaulieu et al., 2007; Knight and Beaulieu, 2008). To account for polyploidy, we calculated the monoploid genome size (1Cx-value) as the 1C-value divided by half the ploidy level (ploidy level/2), which represents the DNA content per basic chromosome set (Greilhuber et al., 2006). This approach enables us to distinguish adaptive significance in genome size–trait relationships from artifacts introduced by variation in ploidy, allowing for a clearer understanding of the evolutionary basis of genome architecture.

Given these complexities, a holistic framework that integrates interaction effects between key axes of diversity, systematically examines variation in genome size–trait relationships, and accounts for phylogenetic non-independence is needed to advance our understanding of plant genome evolution. In this study, we compile an extensive dataset comprising 2,285 angiosperm species, including information on genome size, life cycle, monocot-dicot classification, and multiple functional traits. By jointly analyzing these factors within phylogenetically informed models, we aim to clarify how life history strategies, taxonomic lineage, and evolutionary history interact to shape genome size and its functional correlates. This integrated approach promises to advance our understanding of the adaptive and evolutionary processes underlying angiosperm diversity, with important implications for predicting plant responses to environmental change and guiding biodiversity conservation. Specifically, we address three key questions: (1) How do interactions between life cycle and monocot-dicot distinctions influence genome size variation? (2) To what extent do genome size–trait correlations differ or reverse across functional groups? and (3) Which correlations persist after phylogenetic correction, indicating adaptive significance? Through combining comparative trait analyses with phylogenetically explicit models, we seek to elucidate the synergistic drivers of angiosperm genome evolution.

## Materials and methods

### Source of genome size and functional trait data

This study defined genome size as the DNA 1C value of a species (Greilhuber et al., 2006). The DNA 1C value data is sourced from the latest version of the Plant DNA C-values Database, release 7.1, https://cvalues.science.kew.org/). The Plant DNA C-value database is currently the most complete plant genome size database, which includes genome size data for 10770 species (including subspecies) of angiosperms (Leitch et al., 2019). When a species has multiple genome size data, we use the median of these data to represent the genome size of the species.

The ploidy level data for each plant species were primarily sourced from the latest version of the Plant DNA C-values Database. For species lacking ploidy information in this database, additional data were obtained from The Chromosome Counts Database (CCDB, version 1.66, https://ccdb.tau.ac.il/) and The Ploidy Level Database (PloiDB, https://ploidb.tau.ac.il/). For diploid species, the 1Cx-value is equivalent to the 1C-value. Because many species have a range of ploidy, which can confound the calculation of the monoploid genome size, we used the most frequently cited value in the literature. For polyploids, the 1Cx-value was calculated by dividing the 1C-value by half the ploidy level (i.e., for a tetraploid, 1Cx = 1C/2; for a hexaploid, 1Cx = 1C/3; and so on). Totally, the dataset in this study includes 1,708 diploid species, 376 tetraploids, 106 hexaploids, and 95 species with other ploidy levels (**Supplementary Data 1**).

The life cycle, monocot-dicot distinction, and plant functional traits of species used in this study are mainly sourced from the Flora of China (http://www.iplant.cn/frps) and Plants of the World Online (https://powo.science.kew.org/). Our data collection focused on plant height and the length and width of leaves, flowers, fruits, and seeds for plants with known genome sizes. When the database provided a height range, we used the average of that range as the representative plant height for the species. When recording data on leaf length and width, compound leaves record the length and width of individual leaflets. If the seed or fruit is round or spherical, the length and width values of the trait can be recorded as the same. When calculating the length of pedicels and sepals, if the sample is dioecious, calculate the average of the two to obtain the average length of pedicels and sepals for the plant. When petals are divided into flag petals, wing petals, and keel petals, the average length of these three types of petals should also be calculated to more accurately describe the morphological characteristics of the plant. We identified research subjects by selecting species with known genome sizes that also had available data on life cycle, monocot-dicot distinction, and at least one additional functional trait. This resulted in a total of 2,285 angiosperms spanning 186 families and 940 genera (**Supplementary Data 1**).

### Data analyses

One-way analysis of variance (ANOVA) was used to compare the differences in mean species values of leaf functional traits between annual and perennial angiosperm species and between monocotyledonous and dicotyledonous species. Two-way ANOVA was used to analyze the effects of life cycle, monocot-dicot distinction and their interactions on functional traits. Principal component analysis (PCA) was used to analyze the correlations between plant functional traits and the distributions of the 2,285 species along the PCA axes. The bivariate trait relationships were analyzed with Pearson’s correlation. To meet the normality assumption, the raw data for assessing the relationships between functional traits were log-transformed before analysis. Statistical analyses were conducted using SPSS software (version 16.0; SPSS Inc.).

To account for phylogenetic non-independence among species in trait correlation analyses, we employed ​Phylogenetic Generalized Least Squares (PGLS) for evolutionary signal correction. A phylogenetic tree was reconstructed using the Phylomatic v3.0 program, following the Angiosperm Phylogeny Group IV (APG IV) classification framework (**Supplementary Data 2**). Due to missing or insufficient phylogenetic data for some species, phylogenetic tree construction and analysis were limited to 1,647 species (72.1% of the original dataset). These 1,647 species represent 175 families, accounting for 94.1% of the total 186 families in the original dataset, thereby ensuring that the phylogenetic tree provides sufficient taxonomic representation (**Supplementary Data 2**). The excluded families (Cleomaceae, Linderniaceae, Lecythidaceae, Hernandiaceae, Moringaceae, Paulowniaceae, Dipterocarpaceae, Torricelliaceae, Musaceae, Hypoxidaceae, Xyridaceae) do not disproportionately represent early-diverging monocots or other critical clades that would bias phylogenetic corrections. The proportions of annuals (17.9% vs. 16.3%), perennials (82.1% vs. 83.7%), monocots (30.3% vs. 30.4%), and dicots (69.7% vs. 69.6%) were nearly identical between the full and pruned datasets. Phylogenetic comparative analyses were conducted in R (v4.3.1) using the geiger and phytools packages.

To estimate the evolutionary rates of monoploid genome size (1Cx-value) variation across different functional groups (e.g., annual vs. perennial, monocots vs. dicots), we employed a Brownian Motion (BM) model within a phylogenetic comparative framework. The analysis was conducted using the pruned phylogenetic tree (1,647 species) which was also utilized in the PGLS analyses to ensure methodological consistency. Under the BM model, trait evolution is modeled as a random walk process, where the variance of the trait (σ², the evolutionary rate parameter) accumulates proportionally with time along the branches of the tree. We fitted the BM model to log-transformed 1Cx-values using the fitContinuous function in the R package geiger, which estimates σ² via maximum likelihood. We then tested whether evolutionary rates differed significantly among these groups using a likelihood ratio test (LRT). This involved comparing a single-rate model (where all groups share the same σ²) against a multi-rate model (where each group has an independently estimated σ²). A significant improvement in model fit (*p* < 0.05) for the multi-rate model would indicate divergent evolutionary rates among groups.

## Results

### The interaction of life cycle and monocot/dicot status is the primary driver of genome size variation in angiosperms

Analysis of 2,285 angiosperm species (spanning 186 families and 940 genera) revealed that both life cycle (annual vs. perennial) and monocot/dicot affiliation have significant effects on genome size, whether considered independently or jointly (**Tables 1**, **2**). Perennial species have significantly larger DNA amount 1C (4045 ± 178 Mbp) and DNA amount 1Cx (2541 ± 157 Mbp) than annual species (**Table 1**). Similarly, monocot species exhibit significantly higher DNA amount 1C (7651 ± 408 Mbp) and DNA amount 1Cx (2099 ± 96 Mbp) compared to dicot species (**Table 1**). Furthermore, the interactive effect of life cycle and monocot/dicot status was stronger for DNA amount 1Cx (F = 16.3, *p* < 0.001) than for DNA amount 1C (F = 4.3, *p* < 0.05) (**Table 2**).

**Table 1:** Genome size and organ functional traits (mean ± standard error) of 2,285 angiosperm species with different life form and monocot-dicot distinction.

Functional traits	Units	Life cycle		Monocot-dicot distinction	
		Annual species (399)	Perennial species (1886)	Monocotyledons (693)	Dicotyledons (1592)
DNA Amount 1C	Mbp	2541 ± 157	4045 ± 178 ***	7651 ± 408	2099 ± 96***
DNA Amount 1Cx	Mbp	1830 ± 101	3335 ± 164 ***	6167 ± 377	1726 ± 89***
Plant height	m	0.7 ± 0.1	4.2 ± 0.2***	1.5 ± 0.1	4.5 ± 0.2***
Petiole length	cm	4.8 ± 0.4	7.2 ± 0.4 ns	34.2 ± 1.6	4.1 ± 0.2***
Leaf length	cm	9.8 ± 0.6	14.8 ± 0.8**	29.3 ± 2.0	8.2 ± 0.2***
Leaf width	cm	2.9 ± 0.2	4.5 ± 0.3*	4.7 ± 0.9	4.0 ± 0.1***
Calyx length	cm	0.7 ± 0.1	1.0 ± 0.0*	1.9 ± 0.1	0.7 ± 0.0***
Corolla length	cm	1.9 ± 0.1	2.9 ± 0.1***	4.2 ± 0.1	2.6 ± 0.1**
Petal length	cm	0.9 ± 0.1	1.7 ± 0.1**	2.4 ± 0.2	1.3 ± 0***
Fruit length	cm	2.9 ± 0.5	2.7 ± 0.1 ns	1.7 ± 0.1	3.0 ± 0.2***
Fruit width	cm	0.9 ± 0.1	1.3 ± 0.0 **	1.3 ± 0.1	1.2 ± 0.0 ns
Seed length	mm	3.4 ± 0.2	5.4 ± 0.2*	6.0 ± 0.6	4.7 ± 0.2 ns
Seed width	mm	2.8 ± 0.1	5.0 ± 0.1*	5.9 ± 0.3	4.2 ± 0.1*

*p < 0.05; **p < 0.01; ***p < 0.001; ns, *p* > 0.05.

**Table 2:** Impact of life form and monocot-dicot distinction on genome size and organ functional traits in 2,285 angiosperm species.

Functional traits	Life cycle		Monocot-dicot distinction		Life cycle*monocot-dicot distinction	
	F	*P*	F	*P*	F	*P*
DNA Amount 1C	21.2	***	60.3	***	4.3	*
DNA Amount 1Cx	26.5	***	74.3	***	16.3	***
Plant height	50.2	***	15.1	***	18.9	***
Petiole length	19.2	***	30.6	***	18.5	***
Leaf length	19.1	***	57.5	***	8.5	**
Leaf width	5.1	*	0.1	ns	1.4	ns
Calyx length	1.40	ns	0.11	ns	0.86	ns
Corolla length	6.4	*	0.0	ns	2.6	ns
Petal length	10.4	***	0.2	ns	4.0	*
Fruit length	1.3	ns	15.0	***	7.8	**
Fruit width	12.6	***	2.2	ns	3.8	ns
Seed length	5.0	*	1.2	ns	0.6	ns
Seed width	6.3	*	0.2	ns	1.8	ns

*p < 0.05; **p < 0.01; ***p < 0.001; ns, *p* > 0.05.

**Figure 1:**
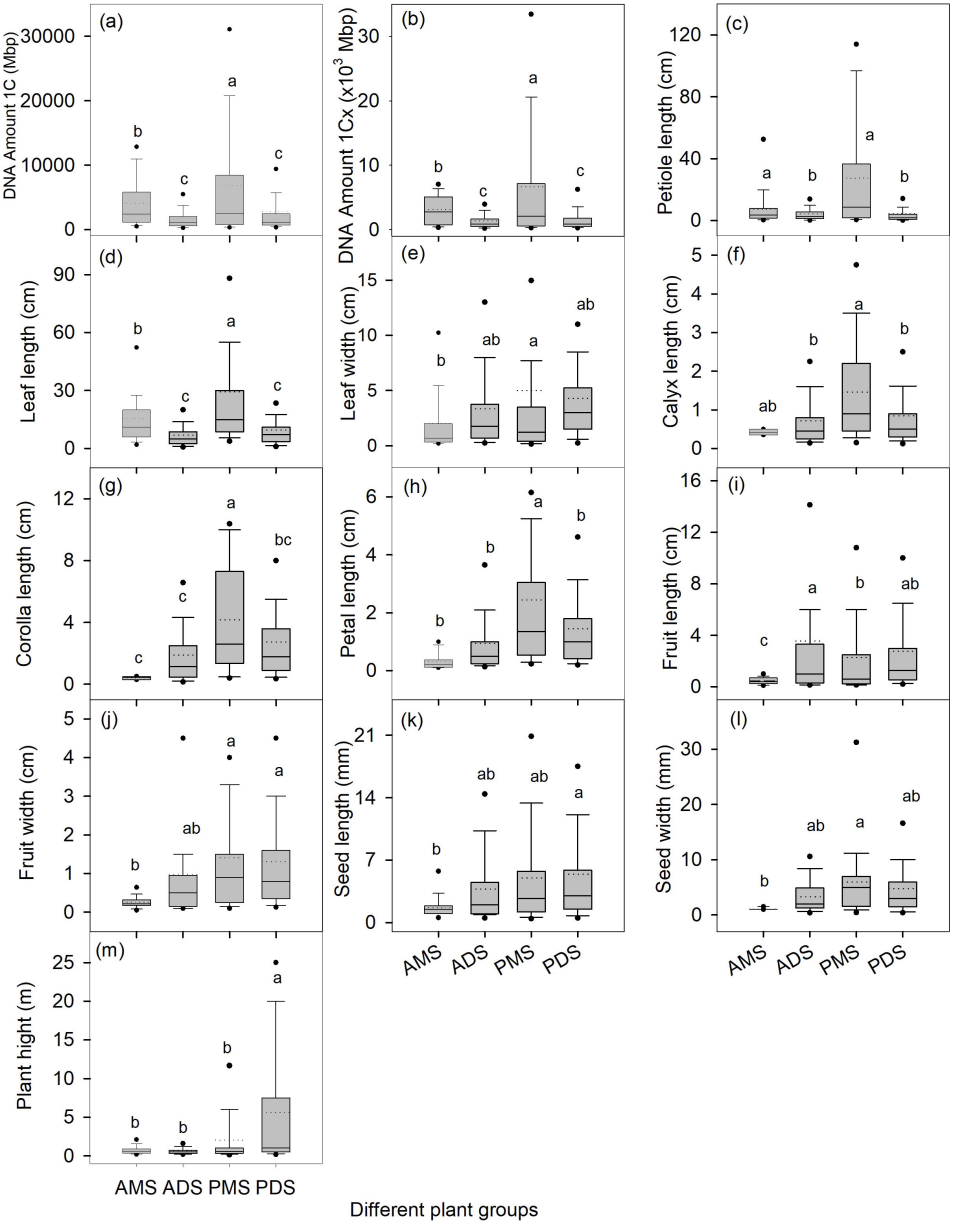
Genome size **(a, b)** and organ functional traits **(c-m)** for annual and perennial species and for monocotyledonous and dicotyledonous species. The box plots depict the variation among different leaf forms and habits, with the bottom and top of the boxes indicating the 25th and 75th percentiles, respectively; the two whiskers represent the 10th and 90th percentiles; the two black points represent the 5th and 95th percentiles; the horizontal lines within the boxes represent the median values, and the dotted lines within the boxes represent the mean values. Different lowercase letters in each panel indicate significant differences in the average values among different groups (one-way ANOVA, *p* < 0.05). AMS, annual monocotyledonous species; ADS, annual dicotyledonous species; PMS, perennial monocotyledonous species;and PDS, perennial dicotyledonous species.

Further dividing all species into four groups (annual monocots/dicots, perennial monocots/dicots) showed that perennial monocots possess the largest DNA amount 1C (8122 ± 470 Mbp) and DNA amount 1Cx (6,688 ± 436 Mbp) (**Figure 1**). The DNA amount (both 1C and 1Cx values) in annual monocots was significantly higher than in both annual and perennial dicots, while there was no significant difference in genome size between the latter two groups (**Figure 1**). In addition to genome size, several morphological traits, such as plant height, petiole length, and leaf length, are also significantly influenced by life cycle, monocot/dicot status, and their interaction (**Table 2**).

### The relationship between genome size and functional traits shows significant group-specific differences and even directional reversals

For all studied species, DNA amount 1C has a significant negative correlation with plant height (*r* = -0.14, *p* < 0.001), while it has a significant positive correlation with petiole length (*r* = 0.11, *p* < 0.001), leaf length (*r* = 0.19, *p* < 0.001), calyx length (*r* = 0.31, *p* < 0.001), corolla length (*r* = 0.14, *p* < 0.001), petal length (*r* = 0.31, *p* < 0.001), fruit width (*r* = 0.12, *p* < 0.001), and seed length (*r* = 0.19, *p* < 0.001). However, there was no relationship between DNA amount 1C and leaf width, fruit length and seed width (**Table 3**). For DNA amount 1Cx, significant negative correlations with plant height were observed across all species (*r* = -0.1, *p* < 0.01) and in perennials (*r* = -0.15, *p* < 0.001), while positive correlations persisted in annuals (*r* = 0.13, *p* < 0.05) (**Table 3**).

**Table 3:** The correlation between genome size and other functional traits of 2,285 species in different plant groups.

Functional traits	DNA amount 1C					DNA amount 1Cx				
	for all	AS	PS	MS	DS	for all	AS	PS	MS	DS
Plant height	-0.14***	0.14*	-0.20***	-0.04	-0.10***	-0.10***	0.13*	-0.15***	-0.05	-0.03
Petiole length	0.11***	0.12	0.11**	-0.24*	0.03	0.06	0.06	0.07	-0.22	-0.03
Leaf length	0.19***	0.22**	0.18***	-0.17**	0.10***	0.18***	0.17***	0.18***	-0.14**	0.13***
Leaf width	-0.02	-0.18***	0.01	0.14***	0.04	0.01	-0.21***	0.04	0.15***	0.063*
Calyx length	0.31***	0.27***	0.30***	0.01	0.22***	0.31***	0.31***	0.30***	0.08	0.21***
Corolla length	0.14***	0.10	0.15***	0.29*	0.08	0.14***	0.12	0.15***	0.27*	0.09*
Petal length	0.31***	0.21*	0.28***	0.31***	0.19***	0.34***	0.22*	0.31***	0.37***	0.20***
Fruit length	0.02	-0.05	0.04	0.43***	-0.07*	0.10***	0.01	0.10***	0.43***	0.01
Fruit width	0.12***	0.08	0.12***	0.31***	0.07	0.13***	0.13	0.12***	0.30***	0.092*
Seed length	0.19***	0.19*	0.17***	0.19	0.20***	0.22***	0.19*	0.20***	0.23	0.23***
Seed width	0.14	0.05	0.11	-0.3	0.19*	0.16*	-0.03	0.15	-0.31	0.21**

*p < 0.05; **p < 0.01; ***p < 0.001. AS, annual species; PS, perennial species; MS, monocotyledonous species; DS, dicotyledonous species.

When species were divided into subgroups, the correlations among different groups not only differ significantly but may even reverse direction. For example, in perennial and dicot species, DNA amount 1C was negatively correlated with plant height (perennials: *r* = -0.20, dicots: *r* = -0.10, both *p* < 0.001); in annual (*r* = 0.14, *p* < 0.05) and annual dicot species (*r* = 0.20, *p* < 0.01), the correlation becomes positive (**Table 3**). In monocots and their subgroups, there was no significant correlation between plant height and DNA amount 1C (**Table 3**; **Supplementary Data 3**, **Supplementary Table S1**). DNA amount 1Cx also showed no correlation with plant height in dicots overall (**Table 3**), but exhibited a positive correlation in annual dicots (*r* = 0.25, *p* < 0.01) and negative correlation in perennial dicots (*r* = -0.13, *p* < 0.01) (**Supplementary Data 3**, **Supplementary Table S1**).

Furthermore, correlations between DNA amount 1C and traits such as leaf length, leaf width, and petiole length also display group specificity. For instance, the positive correlation between petiole length and DNA amount 1C was only observed in perennials and annual monocots, while positive correlations with leaf length and width are mainly found in annual and dicot groups (**Table 3**). For floral organ traits, the positive correlations of both DNA amount 1C and 1Cx with calyx, corolla, and petal length were significant in most groups, especially in perennials and monocots. Correlations of DNA amount 1C and 1Cx with seed or fruit traits are mainly observed in dicot groups (**Table 3**).

**Figure 2:**
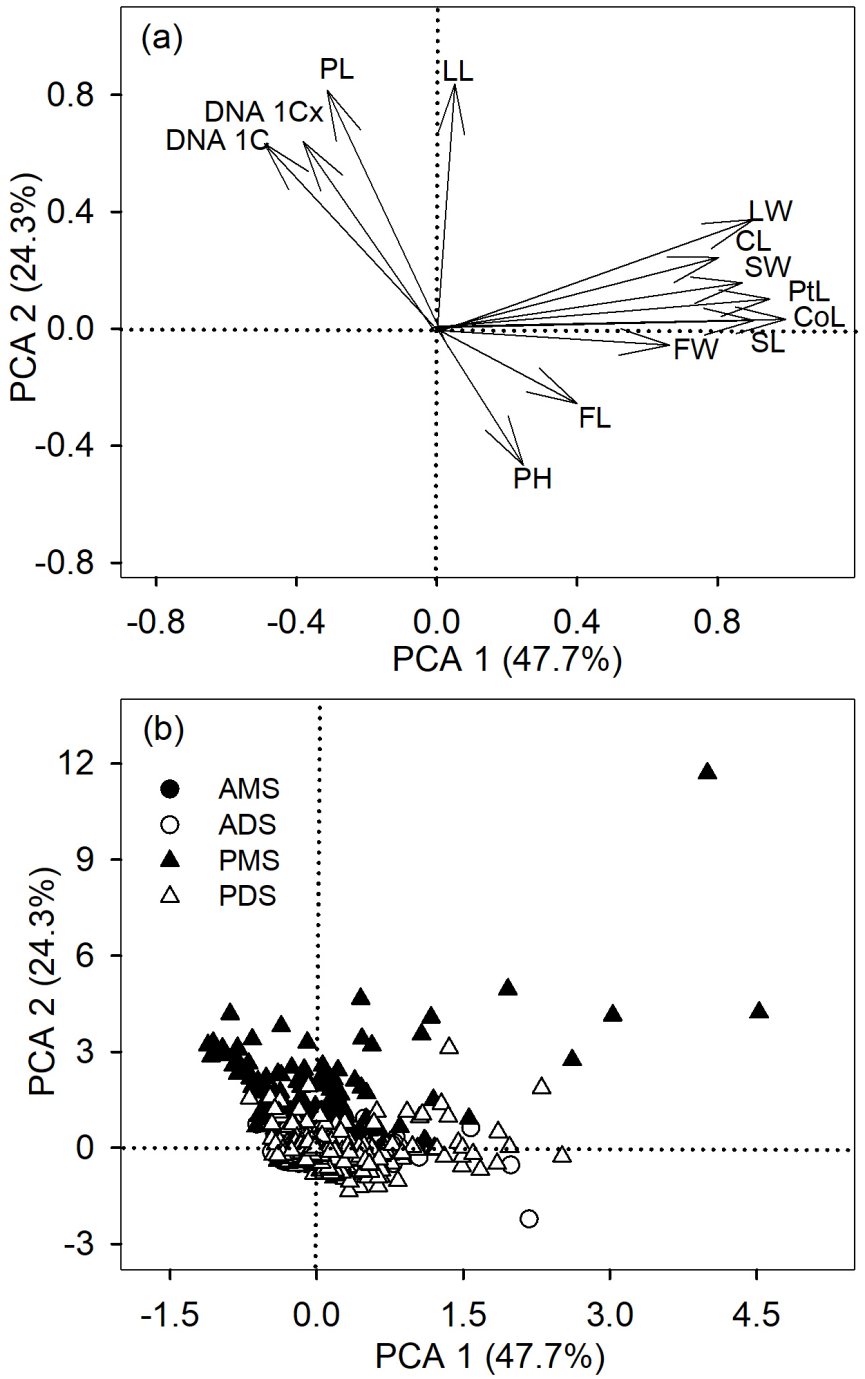
Principal component analysis (PCA) of functional traits across 2,285 angiosperm species. **(a)** Loadings of functional traits on PC1 and PC2, including plant height (PH), petiole length (PL), leaf length (LL), leaf width (LW), calyx length (CL), corolla length (CoL), petal length (PtL), fruit length (FL), fruit width (FW), and seed length (SL). **(b)** The first two PCA axes (PC1 and PC2) showing the distribution of species groups: annual monocotyledonous species (AMS), annual dicotyledonous species (ADS), perennial monocotyledonous species (PMS), and perennial dicotyledonous species (PDS).

Principal component analysis (PCA) further showed that different groups are clearly separated along axes of functional trait variation (**Figure 2**). The first axis of the PCA (PCA 1) accounted for 47.7% of the variance and the second axis (PCA 2) explained 24.3% of the variance among the 13 variables (**Figure 2**). PCA 1 was loaded with corolla length, petal length, and seed length on the positive side, whereas PCA2 was loaded with petiole length, leaf length on the positive side (**Figure 2**). These results demonstrated that the relationship between DNA amount 1C and functional traits was highly dependent on life history and phylogenetic background, and may even exhibit directional reversals, suggesting the presence of distinct adaptive strategies.

### Phylogenetic correction reveals that some genome size–trait correlations disappear while others remain robust across groups

Using phylogenetic generalized least squares (PGLS) regression to account for evolutionary relationships, we found that some previously observed correlations between DNA amount 1C and traits disappeared, suggesting these correlations were primarily due to shared ancestral inheritance. For example, the negative correlation between DNA amount 1C and plant height in perennial and dicot groups became non-significant after phylogenetic correction, indicating that these correlations are mostly constrained by phylogenetic history (**Table 4**). In contrast, the positive correlation between DNA amount 1C and plant height in annual groups remained significant after correction (*r* = 0.19, *p* < 0.01), suggesting adaptive mechanisms independent of phylogenetic history. Regarding petal length, its positive correlation with DNA amount 1C remained significant after phylogenetic correction across all species and groups (e.g., annuals *r* = 0.23, perennials *r* = 0.08, annual monocots *r* = 0.52, all *p* < 0.05), indicating phylogenetically conserved and adaptive significance. By contrast, correlations with seed traits weakened or disappeared after correction, suggesting they are mainly due to phylogenetic history rather than direct adaptive processes.

**Table 4:** Phylogenetically independent contrasts of correlation coefficients between genome size and functional traits of 1,647 species across plant groups.

Functional traits	DNA amount 1C					DNA amount 1Cx				
	for all	AS	PS	MS	DS	for all	AS	PS	MS	DS
Plant height	0.01	0.19**	-0.03	0.0	0.0	-0.001	0.02*	-0.001	0.01*	0.003
Petiole length	-0.04	0.21*	-0.04	-0.03	0.0	0.0	0.02	0.003	0.05	-0.002
Leaf length	0.05*	-0.05	0.03	0.0	0.01**	0.0	-0.003	-0.001	0.01	0.01*
Leaf width	0.06	-0.07	0.06*	0.01	0.0	0.0	0.001	0.001	-0.002	0.0
Calyx length	0.13**	0.19*	0.1*	0.01	0.02**	0.02***	0.04*	0.01**	0.01	0.02**
Corolla length	0.16***	0.22*	0.1*	0.03	0.03***	0.03***	0.06*	0.0	0.03	0.03***
Petal length	0.12**	0.23*	0.08*	0.01	0.01*	0.01**	0.08**	0.01*	0.02*	0.004
Fruit length	0.09**	0.26***	0.02	0.0	0.02***	0.007**	0.08**	0.001	-0.004	0.01***
Fruit width	0.06	0.29**	-0.03	0.0	0.0	-0.001	0.05*	-0.002	-0.01	0.001
Seed length	0.08	0.15	-0.03	0.0	0.02*	0.01*	0.02	0.003	-0.01	0.02**
Seed width	-0.08	-0.17	-0.09	0.06	0.0	-0.003	-0.03	-0.01	0.004	0.004

*p < 0.05; **p < 0.01; ***p < 0.001. AS, annual species; PS, perennial species; MS, monocotyledonous species; DS, dicotyledonous species.

Furthermore, parallel PGLS analyses of monoploid genome size (DNA amount 1Cx)—which accounts for ploidy variation—revealed congruent evolutionary patterns. The directional reversal in plant height correlations persisted: DNA amount 1Cx remained positively correlated with plant height in annuals (*r* = 0.02, *p* < 0.05) and monocots (*r* = 0.01, *p* < 0.05), while the negative correlation observed in perennials became non-significant after phylogenetic correction (**Table 4**; **Supplementary Data 3**, **Supplementary Table S2**). Furthermore, phylogenetic analyses of DNA amount 1Cx demonstrated persistent correlations with floral traits across diverse angiosperm lineages. The PGLS results revealed that DNA amount 1Cx maintained significant positive correlations with calyx length in annual species (*r* = 0.04, *p* < 0.05), perennial species (*r* = 0.01, *p* < 0.01), and dicotyledonous species (*r* = 0.02, *p* < 0.01). Similarly, the petal length also exhibited stable and widespread correlations with DNA amount 1Cx, showing significant positive relationships in annuals (r = 0.08, p < 0.01), perennials (*r* = 0.01, *p* < 0.05), and monocots (*r* = 0.02, *p* < 0.05). These phylogenetically corrected patterns suggest that the fundamental links between genome size and floral morphology represent deep evolutionary constraints rather than phylogenetic artifacts. Conversely, seed traits correlations in nearly all groups became non-significant for both genome size metrics after PGLS, further supporting their phylogenetic dependency (**Table 4**; **Supplementary Data 3**, **Supplementary Table S2**).

### Evolutionary rates of genome size variation

**Figure 3:**
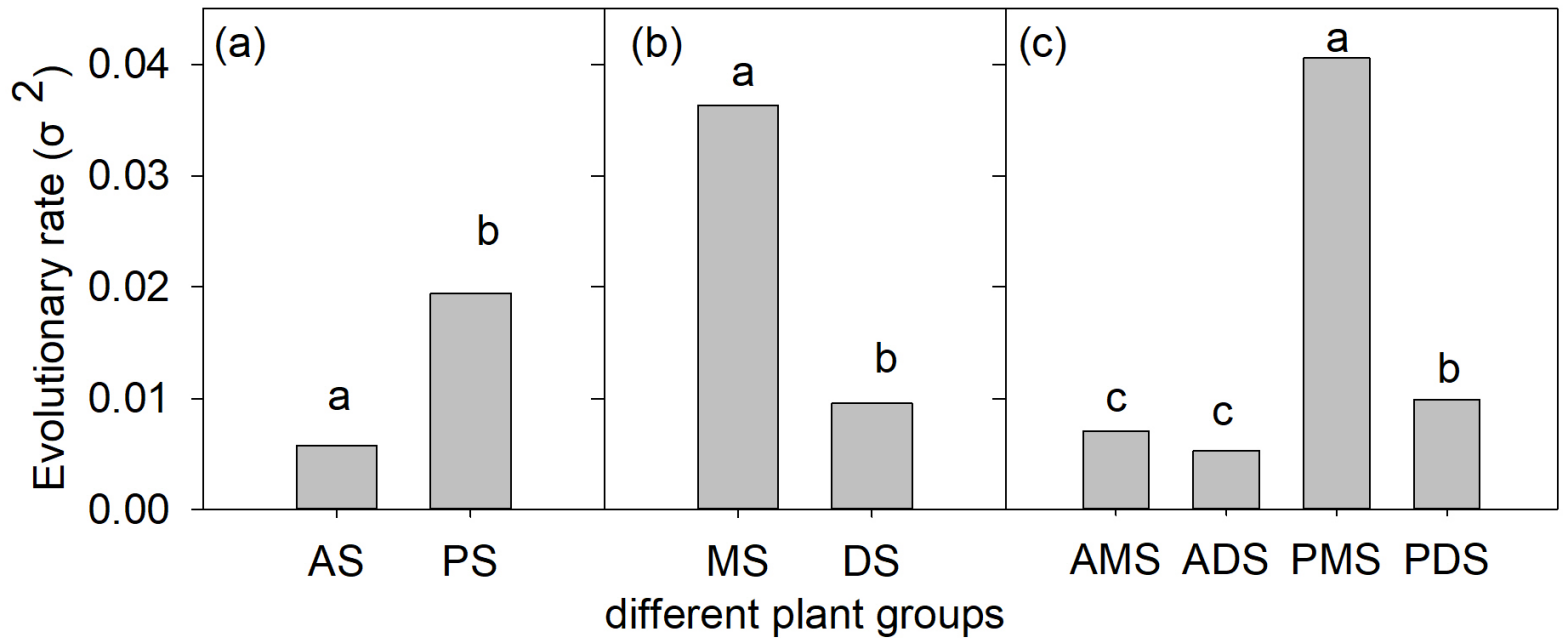
Evolutionary rates (σ²) of monoploid genome size (DNA Amount 1Cx) variation in angiosperms with different life cycle and monocot-dicot lineage **(a-c)**. AS, annual species; PS, perennial species; MS, monocotyledonous species; and DS, dicotyledonous species; AMS, annual monocotyledonous species;ADS, annual dicotyledonous species; PMS, perennial monocotyledonous species;PDS, perennial dicotyledonous species. Different lowercase letters in each panel indicate significant differences in the average values among different groups (*p* < 0.05).

Our analyses revealed significant differences in the evolutionary rates (σ²) of monoploid genome size (1Cx) among functional groups (**Figure 3**). Annual species (σ² = 0.006) exhibited significantly lower evolutionary rates than perennial species (σ² = 0.019; LRT: χ² = 18.9; *p* < 0.001), while monocots (σ² = 0.036) showed higher rates than dicots (σ² = 0.010; LRT: χ² = 362.2; *p* < 0.001). Among the four subdivided groups, perennial monocots (σ² = 0.041) displayed the highest evolutionary rate. Notably, perennial dicots (σ² = 0.010) evolved significantly faster than both annual monocots (σ² = 0.007) and annual dicots (σ² = 0.005), whereas no significant difference was detected between annual monocots and annual dicots (**Figure 3**).

## Discussion

This study integrates two key axes of angiosperm diversity—life cycle (annual vs. perennial) and monocot/dicot classification—combined with phylogenetic comparative analyses, to reveal their profound interactive effects on genome size variation and its relationships with functional traits. The main findings can be summarized as follows: (1) The interactive effects of life cycle and monocot-dicot divergence shape genome size variation in distinct ways: perennial monocots exhibit the largest genomes (1C and 1Cx), whereas genome size does not differ significantly between annual and perennial dicots (**Figure 1**); (2) Genome size–trait correlations are group-specific and may reverse direction (e.g., positive in annuals but negative in perennials for plant height; **Table 3**); (3) Phylogenetic correction shows that some correlations (e.g., the negative correlation between genome size metrics and plant height in perennials) are mainly driven by lineage history, while others (e.g., the positive correlation between genome size metrics and petal length) are robust across lineages, implying adaptive significance.

### Interactive effects shape genome size patterns in angiosperms

Our findings demonstrate that interactive effects between life cycle strategy (perennial vs. annual) and phylogenetic lineage (monocot vs. dicot) are key drivers of genome size variation in angiosperms. Notably, perennial monocots exhibit significantly larger genomes than other groups (**Figure 1**), underscoring the combined influence of longevity and evolutionary history on genome evolution. Long-lived, perennial growth strategies may promote genome expansion by favoring increased cell size and reduced cell division rates, which support persistent structural development and resource allocation across multiple seasons (Kellogg, 2001; Tank and Olmstead, 2008; González-Paleo et al., 2019). In perennial monocots (e.g., Poaceae and Liliaceae), developmental constraints—such as reliance on limited meristematic cell divisions for organogenesis (e.g., grass tillering)—further amplify genome size effects. Larger cell volumes, which correlate positively with increased genome size, may compensate for the limited number of cell divisions, thereby enabling efficient biomass production (e.g., tall stems or large leaves). This developmental constraint-genome size coupling likely underpins the maintenance of ultra-large genomes in these lineages (Winterfeld et al., 2025). Critically, large genomes impose developmental constraints that preclude rapid life cycles (Bennett and Leitch, 2005), making genome size reduction a prerequisite for shifts to annual strategies—as evidenced in lineages like Brachyscome. Thus, genome size expansion is less a direct adaptation than a constraint enabling perenniality under evolutionary pressure.

### Group specificity and correlation reversal reveal adaptive divergence

One of the most striking findings is the directional reversal in the relationship between DNA amount 1C and plant height: a positive correlation in annuals, but a negative correlation in perennials (**Table 3**). This clearly reflects fundamentally different selection pressures and trade-off mechanisms under distinct life history strategies. Annuals must complete their entire life cycle within a single growing season; their “fast strategy” (r-strategy) drives the coevolution of small genomes (facilitating rapid cell division) and shorter stature, maximizing reproductive output (Knight et al., 2005; Shao et al., 2021). In contrast, perennials adopt a “slow strategy” (K-strategy), investing in persistent structures such as woody stems (Moeglein et al., 2020). The negative correlation may arise from the metabolic costs of large genomes limiting resource allocation to supporting structures (e.g., secondary growth) (Grime and Mowforth, 1982; Grime, 1998), or reflect the sustained constraints of large genomes on cell division rates during long-term growth (Simonin and Roddy, 2018). This reversal highlights that ignoring key classification axes (such as life cycle) could lead to partial or even erroneous interpretations of genome size–trait relationships (Guo et al., 2024b).

### Phylogenetic correction reveals the interplay of adaptation and history

PGLS analysis effectively distinguishes phylogenetic conservatism from independent adaptive evolution (**Table 4**). The negative correlation between DNA amount 1C and plant height in perennials and dicots disappears after phylogenetic correction, indicating that this pattern is mainly due to legacy effects from common ancestors (phylogenetic signal) rather than independent adaptive evolution. In contrast, the positive correlation between DNA amount 1C and plant height in annuals remains robust after PGLS, supporting its direct adaptive value in rapid growth strategies. Notably, the positive correlation between DNA amount 1C and petal length remains significant after phylogenetic correction across most of the analysis groups (overall, annual, perennial, dicot; **Table 4**). This strong cross-lineage conservation suggests a deep developmental or functional constraint. For instance, the conserved correlation between genome size and petal length across lineages implies a fundamental link between genomic content and floral morphology, possibly driven by developmental gene networks (Conklin et al., 2019). As a key floral organ, petal size directly influences pollination efficiency (such as display area and nectar position). The increase in cell volume caused by larger genomes may directly determine the minimum number and/or size of petal cells, thereby developmentally coupling genome size with petal length (Nagahama and Yahara, 2019). Beyond developmental constraints, these conserved correlations likely reflect adaptive evolution under pollinator-mediated selection. For instance, in *Paphiopedilum* orchids, larger genomes correlate with increased labellum epidermal cell size and extended floral longevity—a trait directly influencing pollination efficiency (Zhang and Zhang, 2021). Such findings align with broader evidence that floral organs experience distinct selection regimes favoring larger cell sizes compared to leaves, due to relaxed metabolic constraints (Roddy et al., 2020).

In addition to total nuclear DNA content (1C-value), our analyses incorporated monoploid genome size (DNA amount 1Cx) to disentangle the effects of polyploidy from true differences in genome size per basic chromosome set. Our results show that patterns observed for 1Cx largely mirror those for 1C-value, including the strong influence of life cycle and monocot-dicot interactions on genome size variation. This is consistent with previous studies demonstrating that genome size-trait relationships, such as those involving seed mass, can persist even after accounting for ploidy (Beaulieu et al., 2007). Notably, the group-specific and even directionally reversed correlations between genome size and traits such as plant height and floral organ dimensions are robust when using 1Cx, and often persist after phylogenetic correction. For example, the positive association between genome size and petal length remains significant across major groups for both 1C and 1Cx, highlighting a deeply conserved developmental constraint (Bennett and Leitch, 2011). Conversely, some previously reported correlations, such as between genome size and seed traits, have been shown in large-scale studies to have a phylogenetic component and may weaken or disappear after phylogenetic correction, as also observed in our results (Beaulieu et al., 2007). Overall, the use of 1Cx underscores that the observed genome size-trait relationships in angiosperms reflect fundamental evolutionary and adaptive mechanisms rather than simply the effects of polyploidy (Greilhuber et al., 2006; Bennett and Leitch, 2005; Suda et al., 2015).

### Genome size evolutionary rate and its implications for trait associations

Our analyses reveal striking disparities in genome size evolutionary rates (σ²) among angiosperm functional groups, with perennial monocots (PMS) exhibiting the highest rate (σ² = 0.041; **Figure 3**). Contrary to expectations, this accelerated evolution did not correspond to stronger adaptive trait correlations after phylogenetic correction. For instance, while raw data showed a robust association between monoploid genome size (1Cx) and petal length in PMS (*r* = 0.37, *p* < 0.001; **Supplementary Table S1**), PGLS analyses attenuated this relationship (*r* = 0.019, *p* < 0.05; **Supplementary Table S2**), suggesting that shared ancestry—rather than directional selection—primarily drives this pattern. Intriguingly, the inverse pattern emerged in annual dicots (ADS): despite their low evolutionary rate (σ² = 0.005), ADS retained significant phylogenetically independent correlations for traits like calyx length (*r* = 0.04, *p* < 0.05; **Supplementary Table S2**). This decoupling of evolutionary tempo from trait adaptation underscores the multifactorial controls on genome size–trait relationships, where lineage-specific constraints (e.g., developmental canalization in PMS) and ecological strategies (e.g., rapid life cycles in ADS) interact to shape observed patterns (Ackerly, 2009; Malerba et al., 2020).

These results compel a refined framework for interpreting genome size evolution: neither high evolutionary rates nor persistent trait correlations alone suffice to infer adaptation. Instead, integration of rate heterogeneity (σ²), phylogenetic correction, and functional trait analysis is essential to disentangle neutral drift from adaptive evolution. Future studies should prioritize branch-specific models (e.g., OU processes) to identify nodes with elevated selection pressures, coupled with genome-content analyses to pinpoint mechanisms linking DNA content to phenotypic divergence.

### Limitations and future directions

While our study provides comprehensive analysis of morphological trait relationships with genome size, we acknowledge the important limitation of not including key physiological traits such as photosynthetic rate and stomatal conductance, which are known to be influenced by genome size. Future studies could productively integrate resources like the TRY Plant Trait Database to examine how genome size influences both structural and physiological traits simultaneously. Such integration would provide a more complete understanding of genome size’s functional consequences across different organizational levels. In addition, incorporating genome content into the analytical framework may help further unravel the causes and functional consequences of genome size variation.

## Conclusion

In conclusion, by integrating a multidimensional classification framework with phylogenetic methods, this study reveals that the interactive effects of life cycle and monocot/dicot status are key to understanding genome size variation and its functional associations in angiosperms. Furthermore, our use of monoploid genome size as an additional metric confirmed that the observed patterns are not solely driven by polyploidy, but reflect fundamental relationships between genome architecture and plant functional adaptation. Critically, while phylogenetically corrected correlations suggest deep conservation, evolutionary rate heterogeneity reveals that these relationships arise from lineage-specific constraints or selection regimes—not uniform adaptation. This elucidates how apparent correlations may mask underlying adaptive divergence alongside deep conservative mechanisms.

## Data Availability

The original contributions presented in the study are included in the article/**Supplementary Material**. Further inquiries can be directed to the corresponding author.
