# Supplementary material for: Interactive effects of life cycle and monocot-dicot lineage on genome size–trait relationships in angiosperms: a phylogenetically informed analysis
Source: Front Plant Sci. 2025 Aug 29;16:1647198. doi: 10.3389/fpls.2025.1647198 (PMC12426603; doi:10.3389/fpls.2025.1647198)
Supplement: Supplementary file 1 [file Table1.docx]

**Supporting Information**

**Table S1** The correlation between genome size and other functional traits of 2285 species in different plant groups.

| Functional traits | DNA 1C | | | |  | DNA 1Cx | | | |
| --- | --- | --- | --- | --- | --- | --- | --- | --- | --- |
|  | AMS | ADS | PMS | PDS | AMS | | ADS | PMS | PDS |
| Plant height | 0.02 | .23*** | -0.04 | -.22*** | -0.09 | | .25*** | -0.04 | -.13*** |
| Petiole length | 0.98 | 0.04 | -.27* | 0.04 | 0.98 | | -0.04 | -.26* | -0.02 |
| Leaf length | -0.1 | 0.06 | -.18*** | .10** | -0.10 | | 0.1 | -.15** | .14*** |
| Leaf width | -0.2 | 0.00 | .17*** | 0.04 | -.26* | | -0.1 | .19*** | .078* |
| Calyx length | / | .27** | 0.0 | .21*** | / | | .32*** | 0.05 | .18*** |
| Corolla length | 0.95 | 0.13 | .31* | 0.07 | 0.75 | | 0.16 | .30* | 0.08 |
| Petal length | / | .21* | .31*** | .15*** | / | | .23* | .37*** | .16*** |
| Fruit length | .60*** | -0.01 | .44*** | -.09** | .53*** | | 0.06 | .44*** | -0.01 |
| Fruit width | 0.4 | .18* | .36*** | 0.04 | 0.42 | | .24** | .33*** | 0.05 |
| Seed length | -0.1 | .20* | 0.19 | .18*** | -0.69 | | .20* | 0.23 | .21*** |
| Seed width | / | 0.05 | -0.30 | .18* | / | | -0.03 | -0.31 | .227* |

**p* < 0.05; ***p* < 0.01; ****p* < 0.001. AMS = annual monocotyledonous species, ADS = annual dicotyledonous species, PMS = perennial monocotyledonous species, PDS = perennial dicotyledonous species

**Table S2** Phylogenetically independent contrasts of correlation coefficients between genome size and functional traits of 1647 species across plant groups.

| Functional  traits | DNA Amount 1C | | | |  | DNA Amount 1Cx | | | |
| --- | --- | --- | --- | --- | --- | --- | --- | --- | --- |
|  | AMS | ADS | PMS | PDS | | AMS | ADS | PMS | PDS |
| Plant height | -0.04 | 0.20** | -0.06 | -0.04 | | -0.01 | 0.05** | 0.01 | -0.001 |
| Petiole length | 0.47* | 0.21* | 0.06 | -0.05 | | 0.91 | 0.001 | 0.07* | -0.002 |
| Leaf length | -0.11 | -0.05 | -0.06 | 0.06 | | 0.003 | -0.002 | 0.001 | 0.004* |
| Leaf width | 0.11 | -0.08 | 0.07 | 0.05 | | 0.02 | -0.005 | 0.00 | 0.00 |
| Calyx length | / | 0.21* | 0.09 | 0.11* | | / | 0.04* | 0.01 | 0.01 |
| Corolla length | / | 0.23* | 0.17 | 0.09* | | 0.13 | 0.06* | 0.037 | 0.01 |
| Petal length | 0.52* | 0.16 | / | 0.04 | | / | 0.08** | 0.019* | -0.002 |
| Fruit length | 0.57*** | 0.29*** | -0.07 | 0.04 | | 0.04 | 0.08*** | -0.005 | 0.003 |
| Fruit width | 0.11 | 0.36*** | 0.11 | -0.05 | | 0.15 | 0.07** | -0.01 | -0.002 |
| Seed length | -0.23 | 0.24* | -0.15 | 0.02 | | -0.21 | 0.02 | / | 0.01 |
| Seed width | / | / | -0.15 | 0.02 | | / | -0.03 | -0.003 | -0.01 |

**p* < 0.05; ***p* < 0.01; ****p* < 0.001. AMS = annual monocotyledonous species, ADS = annual dicotyledonous species, PMS = perennial monocotyledonous species, PDS = perennial dicotyledonous species
