# Supplementary material for: Interactive effects of life cycle and monocot-dicot lineage on genome size–trait relationships in angiosperms: a phylogenetically informed analysis
Source: Front Plant Sci. 2025 Aug 29;16:1647198. doi: 10.3389/fpls.2025.1647198 (PMC12426603; doi:10.3389/fpls.2025.1647198)
Supplement: Supplementary file 2 [file Table2.docx]

((((((((((((((((((((((((((((((((Eupatorium_fortunei:1.078904,Eupatorium_cannabinum:1.078904):7.403814,Stevia_rebaudiana:8.482718):5.128367,Galinsoga_parviflora:13.611085):4.084262,((((Helianthus_tuberosus:1.528499,Helianthus_annuus:1.528499):5.493554,((Ambrosia_artemisiifolia:1.891768,Ambrosia_trifida:1.891768):3.231282,Xanthium_strumarium:5.12305):1.899003):1.925162,Echinacea_purpurea:8.947215):1.396365,(Sphagneticola_trilobata:3.219111,Eclipta_prostrata:3.219111):7.124469):7.351767):0.462661,((((Bidens_pilosa:5.015197,(Bidens_cernua:2.574992,Bidens_frondosa:2.574992):2.440205):0.011127,(Coreopsis_grandiflora:2.674672,Coreopsis_tripteris:2.674672):2.351652):1.200331,(Cosmos_sulphureus:2.308418,Cosmos_bipinnatus:2.308418):3.918237):4.332976,Dahlia_pinnata:10.559631):7.598377):2.744916,(Filago_arvensis:4.139664,((Leontopodium_sinense:1.175403,Leontopodium_artemisiifolium:1.175403):0.034173,Leontopodium_himalayanum:1.209576):2.930088):16.76326):1.196783,(Buphthalmum_salicifolium:7.796839,(Inula_helenium:6.194877,Carpesium_cernuum:6.194877):1.601962):14.302868):0.889753,(((((((Symphyotrichum_ericoides:3.59922,(Solidago_virgaurea:0.301856,Solidago_canadensis:0.301856):3.297364):0.693518,Erigeron_annuus:4.292738):0.564879,Aster_alpinus:4.857617):0.123888,Bellis_perennis:4.981505):13.961597,((((((((((((Artemisia_macilenta:0.010646,Artemisia_oxycephala:0.010646):0.017574,Artemisia_bargusinensis:0.02822):0.005479,Artemisia_campestris:0.033699):0.1435,Artemisia_scoparia:0.177199):0.941607,(Artemisia_dracunculus:0.277847,Artemisia_giraldii:0.277847):0.840959):0.632133,Artemisia_tournefortiana:1.750939):0.039174,Artemisia_annua:1.790113):0.036046,((((Artemisia_frigida:0.873065,(Artemisia_absinthium:0.826145,Artemisia_sieversiana:0.826145):0.04692):0.113878,((Artemisia_princeps:0.648448,(Artemisia_dubia:0.393646,Artemisia_vulgaris:0.393646):0.254802):0.104947,Artemisia_desertorum:0.753395):0.233548):0.109194,(Artemisia_tridentata:1.080989,Artemisia_lagocephala:1.080989):0.015148):0.712219,Artemisia_austriaca:1.808356):0.017803):0.06458,Neopallasia_pectinata:1.890739):0.450457,((((Chrysanthemum_rhombifolium:0.104175,Chrysanthemum_indicum:0.104175):0.056712,Chrysanthemum_potentilloides:0.160887):0.003875,Chrysanthemum_lavandulifolium:0.164762):0.048436,Chrysanthemum_vestitum:0.213198):2.127998):0.521766,Leucanthemella_linearis:2.862962):2.857527,(((((Achillea_setacea:0.296022,Achillea_millefolium:0.296022):0.158755,Achillea_asiatica:0.454777):1.716494,((Achillea_salicifolia:0.209991,Achillea_impatiens:0.209991):0.101974,Achillea_ptarmica:0.311965):1.859306):1.699205,((Tanacetum_cinerariifolium:2.211904,Tanacetum_vulgare:2.211904):0.893825,Anthemis_cotula:3.105729):0.764747):0.805171,((Argyranthemum_frutescens:3.658231,Chamaemelum_nobile:3.658231):0.545648,Leucanthemum_vulgare:4.203879):0.471768):1.044842):13.222613):0.231742,Calendula_officinalis:19.174844):2.273935,(((Senecio_vulgaris:1.057921,Senecio_macranthus:1.057921):0.498048,Senecio_glomeratus:1.555969):11.355865,Tussilago_farfara:12.911834):8.536945):1.540681):7.684679,((((((((Crepis_chrysantha:3.503768,Crepis_sibirica:3.503768):0.339531,(Crepis_crocea:2.159594,Crepis_tectorum:2.159594):1.683705):2.107738,Crepis_multicaulis:5.951037):4.644589,(Picris_hieracioides:6.606473,Hypochaeris_maculata:6.606473):3.989153):1.945636,((Sonchus_asper:3.133129,Sonchus_arvensis:3.133129):4.564982,Reichardia_tingitana:7.698111):4.843151):0.355331,(((Lactuca_sativa:0.415661,Lactuca_serriola:0.415661):2.534743,(Lactuca_tatarica:1.590351,Lactuca_sibirica:1.590351):1.360053):1.619262,Lactuca_indica:4.569666):8.326927):3.677891,((Hieracium_virosum:0.005368,Hieracium_umbellatum:0.005368):14.760298,Cichorium_intybus:14.765666):1.808818):9.204894,(Tragopogon_porrifolius:3.08296,(Tragopogon_dubius:1.660653,Tragopogon_pratensis:1.660653):1.422307):22.696418):4.894761):3.606085,(((((((Centaurea_diffusa:2.461962,Centaurea_nigrescens:2.461962):2.888281,Centaurea_calcitrapa:5.350243):0.560691,Centaurea_cyanus:5.910934):0.353361,(Carthamus_lanatus:2.53801,Carthamus_tinctorius:2.53801):3.726285):5.865799,((Arctium_lappa:9.425099,(((Cirsium_arvense:1.090996,Cirsium_vulgare:1.090996):2.432127,Silybum_marianum:3.523123):1.205325,(Carduus_crispus:1.897734,Carduus_nutans:1.897734):2.830714):4.696651):0.68183,Onopordum_acanthium:10.106929):2.023165):3.708132,Carlina_vulgaris:15.838226):0.131473,((((((Echinops_ritro:1.354775,Echinops_talassicus:1.354775):0.061043,Echinops_setifer:1.415818):0.534928,Echinops_sphaerocephalus:1.950746):0.170683,Echinops_cornigerus:2.121429):3.507213,Echinops_humilis:5.628642):0.699743,Echinops_nanus:6.328385):9.641314):18.310525):4.014047,Gerbera_piloselloides:38.294271):29.975079,Nymphoides_peltata:68.26935):14.933416,((Campanula_glomerata:13.529344,Adenophora_liliifolia:13.529344):22.453845,(Canarina_canariensis:20.130373,Platycodon_grandiflorus:20.130373):15.852816):47.219577)Asterales.rn.d8s.tre:10.527446,(((((((((((Libanotis_spodotrichoma:9.372858,(Heracleum_moellendorffii:6.131328,Pastinaca_sativa:6.131328):3.24153):0.01924,Coriandrum_sativum:9.392098):0.425006,Levisticum_officinale:9.817104):6.685216,(Carum_carvi:16.296178,(Petroselinum_crispum:8.902779,Foeniculum_vulgare:8.902779):7.393399):0.206142):6.030024,(((Daucus_carota:12.57535,Cuminum_cyminum:12.57535):2.48378,(Torilis_japonica:10.660994,Turgenia_latifolia:10.660994):4.398136):1.879238,Anthriscus_sylvestris:16.938368):5.593976):1.071837,Berula_erecta:23.604181):7.868752,(Bupleurum_longicaule:3.279111,Bupleurum_candollei:3.279111):28.193822):11.9949,(Eryngium_planum:27.573815,Astrantia_major:27.573815):15.894018):16.720333,Hydrocotyle_vulgaris:60.188166):3.181033,Pittosporum_tobira:63.369199):22.271456,((((((Valeriana_officinalis:26.81423,Nardostachys_jatamansi:26.81423):4.092454,(Patrinia_scabiosifolia:14.61659,Patrinia_rupestris:14.61659):16.290094):5.868025,((Dipsacus_sativus:2.750064,Dipsacus_fullonum:2.750064):19.039413,Scabiosa_ochroleuca:21.789477):14.985232):4.432354,(Linnaea_borealis:40.122733,Morina_longifolia:40.122733):1.08433):9.544611,(((((((Lonicera_tatarica:2.130595,Lonicera_korolkowii:2.130595):5.56493,Lonicera_ruprechtiana:7.695525):4.25695,(Lonicera_reticulata:9.112814,Lonicera_maackii:9.112814):2.839661):0.59899,Lonicera_nigra:12.551465):0.392997,Lonicera_japonica:12.944462):1.48091,Lonicera_fragrantissima:14.425372):5.052851,Lonicera_sempervirens:19.478223):31.273451):20.186291,((Viburnum_acerifolium:13.08362,Viburnum_opulus:13.08362):45.769661,((Sambucus_racemosa:11.584603,Sambucus_nigra:11.584603):10.443193,Adoxa_moschatellina:22.027796):36.825485):12.084684)Dipsacales.rn.d8s.tre:14.70269)mrcaott1673ott2128:8.089557)mrcaott320ott1673:8.962629,(Ilex_paraguariensis:42.735524,Ilex_cornuta:42.735524):59.957317)campanulids:4.04857,(((((((((((((((((Monarda_fistulosa:5.915268,Mentha_longifolia:5.915268):3.646875,Origanum_vulgare:9.562143):6.270725,Prunella_vulgaris:15.832868):0.673667,(Nepeta_cataria:15.039285,(Hyssopus_officinalis:13.977887,Glechoma_hederacea:13.977887):1.061398):1.46725):1.876015,Melissa_officinalis:18.38255):1.217795,((Salvia_splendens:16.428868,Salvia_glutinosa:16.428868):2.251597,((Salvia_viridis:9.134691,Salvia_nemorosa:9.134691):0.895288,Salvia_officinalis:10.029979):8.650486):0.91988):5.757407,((Ocimum_basilicum:6.48362,Ocimum_tenuiflorum:6.48362):17.205538,Perilla_frutescens:23.689158):1.668594):8.922666,(((Clerodendrum_trichotomum:28.575714,(((Lamium_amplexicaule:8.217074,Lamium_album:8.217074):6.07171,Stachys_sylvatica:14.288784):12.530365,(Scutellaria_baicalensis:0.638288,Scutellaria_viscidula:0.638288):26.180861):1.756565):0.925521,Tectona_grandis:29.501235):1.108328,Vitex_negundo:30.609563):3.670855):6.008518,((((Orobanche_alba:6.781143,Orobanche_caryophyllacea:6.781143):4.637608,Orobanche_cernua:11.418751):22.503118,Lindenbergia_philippensis:33.921869):2.566144,Phryma_leptostachya:36.488013):3.800923):6.982502,(((Acanthus_mollis:43.42085,(((((((Utricularia_australis:1.609383,Utricularia_minor:1.609383):0.835885,Utricularia_vulgaris:2.445268):2.637907,Utricularia_intermedia:5.083175):7.157369,Utricularia_aurea:12.240544):4.459333,Utricularia_gibba:16.699877):12.98691,((Utricularia_graminifolia:16.878984,Utricularia_uliginosa:16.878984):12.438936,Utricularia_caerulea:29.31792):0.368867):3.199951,((Pinguicula_vulgaris:1.523166,Pinguicula_grandiflora:1.523166):10.4598,Pinguicula_gypsicola:11.982966):20.903772):10.534112):3.613818,(((((Parmentiera_cereifera:5.926484,Handroanthus_impetiginosus:5.926484):9.14909,Kigelia_africana:15.075574):25.213827,(Tecoma_stans:27.024122,Campsis_radicans:27.024122):13.265279):2.374436,Jacaranda_mimosifolia:42.663837):3.347511,Sesamum_indicum:46.011348):1.02332):0.129346,Lantana_camara:47.164014):0.107424):1.601973,(Scrophularia_ningpoensis:28.795235,(Buddleja_davidii:2.786816,Buddleja_lindleyana:2.786816):26.008419):20.078176):4.059351,((((((((Veronica_polita:0.601903,Veronica_persica:0.601903):13.529896,((Veronica_chamaedrys:5.60404,Veronica_verna:5.60404):4.043773,Veronica_arvensis:9.647813):4.483986):0.554255,Veronica_ciliata:14.686054):3.546368,((((Veronica_anagallis-aquatica:4.698164,Veronica_beccabunga:4.698164):2.015181,Veronica_peregrina:6.713345):3.37407,Veronica_serpyllifolia:10.087415):7.716197,Veronica_spicata:17.803612):0.42881):12.300965,((Plantago_major:3.913085,Plantago_media:3.913085):12.6955,(Plantago_lanceolata:9.471297,Plantago_ovata:9.471297):7.137288):13.924802):0.122046,Digitalis_purpurea:30.655433):10.708755,(Linaria_vulgaris:22.914909,Antirrhinum_majus:22.914909):18.449279):3.794928,Penstemon_digitalis:45.159116):7.773646):5.166068,((((((((((((Primulina_orthandra:4.938356,Primulina_lutea:4.938356):0.644725,Primulina_eburnea:5.583081):1.017916,(Primulina_chizhouensis:4.350412,Primulina_latinervis:4.350412):2.250585):0.869667,((((Primulina_juliae:2.17473,Primulina_xiuningensis:2.17473):0.936661,Primulina_depressa:3.111391):1.747542,((Primulina_tabacum:3.457804,Primulina_mabaensis:3.457804):1.312174,Primulina_lobulata:4.769978):0.088955):2.565056,(Primulina_roseoalba:2.320164,Primulina_langshanica:2.320164):5.103825):0.046675):0.706902,((((((Primulina_spinulosa:1.309308,Primulina_hedyotidea:1.309308):0.747059,Primulina_ophiopogoides:2.056367):0.280141,Primulina_wentsaii:2.336508):5.144707,Primulina_pseudoeburnea:7.481215):0.283763,(Primulina_linearifolia:2.750047,Primulina_longgangensis:2.750047):5.014931):0.217672,Primulina_huaijiensis:7.98265):0.194916):0.210227,((Primulina_macrodonta:4.604956,Primulina_qingyuanensis:4.604956):0.353919,Primulina_bicolor:4.958875):3.428918):0.00147,((Primulina_mollifolia:7.651097,Primulina_parvifolia:7.651097):0.161533,Primulina_leprosa:7.81263):0.576633):0.390904,(((((Primulina_tribracteata:1.061099,Primulina_liguliformis:1.061099):0.094558,Primulina_sclerophylla:1.155657):0.939662,((Primulina_liboensis:0.576233,Primulina_leiophylla:0.576233):0.180326,Primulina_napoensis:0.756559):1.33876):3.615797,Primulina_ronganensis:5.711116):0.760696,Primulina_renifolia:6.471812):2.308355):0.531498,(((((((((((Primulina_tenuifolia:1.232176,Primulina_lunglinensis:1.232176):0.835408,Primulina_baishouensis:2.067584):0.370566,(Primulina_gueilinensis:1.906197,Primulina_subrhomboidea:1.906197):0.531953):0.190795,(Primulina_shouchengensis:2.133434,Primulina_longii:2.133434):0.495511):0.257569,Primulina_longicalyx:2.886514):0.745698,Primulina_cordata:3.632212):0.642311,(Primulina_glandulosa:3.144494,Primulina_subulata:3.144494):1.130029):0.41465,(((Primulina_fimbrisepala:2.030191,Primulina_verecunda:2.030191):0.594732,Primulina_villosissima:2.624923):0.715195,Primulina_hochiensis:3.340118):1.349055):0.258466,((Primulina_pinnatifida:2.153296,Primulina_obtusidentata:2.153296):0.179978,Primulina_yungfuensis:2.333274):2.614365):0.211078,Primulina_bipinnatifida:5.158717):2.85673,(Primulina_repanda:5.27458,Primulina_cordifolia:5.27458):2.740867):1.296218):0.723732,(Primulina_swinglei:9.362231,Primulina_heterotricha:9.362231):0.673166):3.376283,(((Hemiboea_wangiana:2.251589,Hemiboea_cavaleriei:2.251589):6.144199,Hemiboea_gracilis:8.395788):1.790889,(Lysionotus_sangzhiensis:1.497222,Lysionotus_pauciflorus:1.497222):8.689455):3.225003):0.42039,Aeschynanthus_acuminatus:13.83207):44.26676):12.921779,(((Olea_europaea:17.739301,(Fraxinus_pennsylvanica:1.643279,Fraxinus_americana:1.643279):16.096022):2.37228,(Syringa_vulgaris:5.552195,Ligustrum_quihoui:5.552195):14.559386):11.982465,Nyctanthes_arbor-tristis:32.094046):38.926563):17.227168,(((Cynoglossum_officinale:19.795607,Asperugo_procumbens:19.795607):6.198778,((Echium_vulgare:16.133048,Lithospermum_officinale:16.133048):8.090714,((Anchusa_officinalis:13.562409,Borago_officinalis:13.562409):3.128948,Symphytum_officinale:16.691357):7.532405):1.770623):35.189402,Nemophila_menziesii:61.183787)Boraginales.rn.d8s.tre:27.06399)mrcaott248ott2108:1.503291,((((((Galium_boreale:5.922827,Galium_palustre:5.922827):0.479384,(((Galium_mollugo:0.9831,Galium_verum:0.9831):1.336161,Galium_aparine:2.319261):0.414748,Galium_triflorum:2.734009):3.668202):14.468628,Nertera_granadensis:20.870839):28.87867,(((Catunaregam_spinosa:5.771659,Gardenia_jasminoides:5.771659):1.305947,((Coffea_stenophylla:0.680132,Coffea_arabica:0.680132):2.144865,(Coffea_liberica:2.665108,Coffea_congensis:2.665108):0.159889):4.252609):20.947517,(Adina_pilulifera:17.677149,Hamelia_patens:17.677149):10.347974):21.724386):17.978354,(((((Asclepias_incarnata:16.93923,(Ceropegia_woodii:16.037748,Hoya_carnosa:16.037748):0.901482):4.955229,(Holarrhena_pubescens:12.196276,Wrightia_coccinea:12.196276):9.698183):9.000046,(Catharanthus_roseus:13.639829,(Vinca_major:1.108451,Vinca_minor:1.108451):12.531378):17.254676):21.178612,(Gentiana_macrophylla:2.947982,Gentiana_kurroo:2.947982):49.125135):7.974388,Strychnos_nux-vomica:60.047505):7.680358)Gentianales.rn.d8s.tre:18.185274,(((((((((Solanum_melongena:6.442444,Solanum_torvum:6.442444):1.046889,Solanum_mammosum:7.489333):2.564361,Solanum_pseudocapsicum:10.053694):2.460443,((Solanum_dulcamara:10.547315,(Solanum_aviculare:3.38016,Solanum_laciniatum:3.38016):7.167155):1.083944,Solanum_lycopersicum:11.631259):0.882878):5.88232,(Withania_somnifera:17.56658,Capsicum_annuum:17.56658):0.829877):0.376757,(Datura_stramonium:2.348697,Datura_innoxia:2.348697):16.424517):1.411879,(((Lycium_chinense:1.301561,Lycium_barbarum:1.301561):0.439332,Lycium_ruthenicum:1.740893):14.690371,Hyoscyamus_niger:16.431264):3.753829):9.727617,(((Nicotiana_tabacum:8.258122,Nicotiana_alata:8.258122):0.923079,Nicotiana_glauca:9.181201):2.274349,Nicotiana_rustica:11.45555):18.45716):36.737115,((((((Ipomoea_quamoclit:5.680169,(Ipomoea_nil:3.297676,Ipomoea_purpurea:3.297676):2.382493):1.584582,Ipomoea_batatas:7.264751):0.949739,Ipomoea_aquatica:8.21449):1.629343,Ipomoea_obscura:9.843833):6.407823,(Convolvulus_arvensis:14.579535,Calystegia_sepium:14.579535):1.672121):30.731548,(Cuscuta_europaea:42.236514,Cuscuta_lupuliformis:42.236514):4.74669):19.666621):19.263312)mrcaott1191ott2192:3.837931)mrcaott248ott1191:12.636506,(Aucuba_japonica:43.547103,Eucommia_ulmoides:43.547103)Garryales.rn.d8s.tre:58.840471)lamiids:4.353837)mrcaott248ott320:5.599318,(((((Empetrum_nigrum:26.848983,Vaccinium_myrtillus:26.848983):19.294533,((Pyrola_rotundifolia:18.911145,Orthilia_secunda:18.911145):2.230919,((Moneses_uniflora:0.244844,Monotropa_uniflora:0.244844):16.364126,Chimaphila_umbellata:16.60897):4.533094):25.001452):44.219122,((((Actinidia_eriantha:7.848479,Actinidia_chinensis:7.848479):2.726112,Actinidia_arguta:10.574591):1.517841,Actinidia_kolomikta:12.092432):0.92756,Actinidia_polygama:13.019992):77.342646):4.520655,((((Primula_veris:40.60613,(Ardisia_crenata:13.536873,Myrsine_africana:13.536873):27.069257):46.348468,(Diospyros_montana:8.954405,(Diospyros_lotus:5.401541,Diospyros_kaki:5.401541):3.552864):78.000193):7.403593,(Madhuca_longifolia:61.911799,((((((((Camellia_petelotii:4.28926,((((Camellia_crassicolumna:0.323871,Camellia_tachangensis:0.323871):2.176592,Camellia_luteoflora:2.500463):0.255459,Camellia_rhytidocarpa:2.755922):0.682413,(Camellia_anlungensis:1.513007,Camellia_parvimuricata:1.513007):1.925328):0.850925):0.307756,Camellia_yunnanensis:4.597016):0.177581,Camellia_impressinervis:4.774597):1.631122,(((Camellia_tuberculata:5.523627,(Camellia_pitardii:1.9344,Camellia_reticulata:1.9344):3.589227):0.363574,((Camellia_crapnelliana:1.855707,Camellia_hongkongensis:1.855707):1.211778,Camellia_pubifurfuracea:3.067485):2.819716):0.371462,((((Camellia_transarisanensis:3.146122,Camellia_costei:3.146122):0.780821,(Camellia_fraterna:2.703635,Camellia_lawii:2.703635):1.223308):0.232225,Camellia_salicifolia:4.159168):0.288555,Camellia_sasanqua:4.447723):1.81094):0.147056):0.222076,(((((((Camellia_brevistyla:1.849676,Camellia_fluviatilis:1.849676):1.761291,Camellia_oleifera:3.610967):0.992023,(Camellia_uraku:1.239409,Camellia_japonica:1.239409):3.363581):0.103139,(Camellia_edithae:3.70703,Camellia_azalea:3.70703):0.999099):0.165856,Camellia_chekiangoleosa:4.871985):0.47349,Camellia_semiserrata:5.345475):0.255857,Camellia_granthamiana:5.601332):1.026463):0.314923,((Camellia_saluenensis:3.446084,(((Camellia_mairei:1.624618,Camellia_synaptica:1.624618):0.051796,Camellia_polyodonta:1.676414):1.422231,Camellia_grijsii:3.098645):0.347439):0.606043,Camellia_subintegra:4.052127):2.890591):0.190048,(((Camellia_leptophylla:1.373421,Camellia_ptilophylla:1.373421):1.174142,Camellia_sinensis:2.547563):0.073951,Camellia_grandibracteata:2.621514):4.511252):0.785126,(Camellia_kwangsiensis:2.343978,Camellia_taliensis:2.343978):5.573914):53.993907):32.446392):0.303659,(Phlox_paniculata:29.804278,Polemonium_reptans:29.804278):64.857572):0.221443):8.693903,(Impatiens_balsamina:29.884459,Impatiens_parviflora:29.884459):73.692737)Ericales.rn.d8s.tre:8.763533)mrcaott248ott650:2.225571,((((Hydrangea_integrifolia:11.207761,Hydrangea_anomala:11.207761):5.763616,Hydrangea_aspera:16.971377):8.811595,(Hydrangea_macrophylla:22.367108,(Hydrangea_paniculata:2.724248,Hydrangea_heteromalla:2.724248):19.64286):3.415864):72.204593,((Cornus_sanguinea:8.908743,Cornus_sericea:8.908743):38.801684,(Cornus_canadensis:46.302533,Cornus_officinalis:46.302533):1.407894):50.277138):16.578735)mrcaott248ott27233:5.307934,((((((((Carnegiea_gigantea:14.277927,(Mammillaria_hahniana:9.155023,Astrophytum_ornatum:9.155023):5.122904):7.291258,Opuntia_dillenii:21.569185):17.946199,(Anredera_cordifolia:18.485663,Basella_alba:18.485663):21.029721):33.593404,Mollugo_verticillata:73.108788):9.846651,(Mesembryanthemum_crystallinum:74.876157,((Mirabilis_jalapa:43.01014,(Bougainvillea_spectabilis:6.869391,Bougainvillea_glabra:6.869391):36.140749):28.430675,Phytolacca_americana:71.440815):3.435342):8.079282):4.895165,(((((((Silene_otites:11.172184,(Silene_vulgaris:3.940123,Silene_pendula:3.940123):7.232061):12.722905,Agrostemma_githago:23.895089):18.872736,(Dianthus_caryophyllus:28.452142,Saponaria_officinalis:28.452142):14.315683):8.052007,((Arenaria_serpyllifolia:33.06261,Moehringia_lateriflora:33.06261):12.299723,((Cerastium_fontanum:2.286891,Cerastium_arvense:2.286891):14.058263,(Stellaria_graminea:10.688744,Stellaria_media:10.688744):5.65641):29.017179):5.457499):1.610065,(Spergularia_rubra:38.170756,Spergula_arvensis:38.170756):14.259141):6.451735,Herniaria_glabra:58.881632):11.231,((Salicornia_europaea:40.249417,((((((Atriplex_patula:0.48687,Atriplex_prostrata:0.48687):0.248457,Atriplex_hortensis:0.735327):0.804262,Atriplex_nummularia:1.539589):6.641005,((Chenopodium_album:0.802158,Chenopodium_ficifolium:0.802158):0.084894,Chenopodium_giganteum:0.887052):7.293542):13.835793,Spinacia_oleracea:22.016387):12.899725,Beta_vulgaris:34.916112):5.333305):3.411909,(((((((((Amaranthus_cruentus:2.11455,Amaranthus_spinosus:2.11455):0.390223,(Amaranthus_hypochondriacus:1.26024,Amaranthus_caudatus:1.26024):1.244533):0.842808,Amaranthus_retroflexus:3.347581):0.647154,Amaranthus_hybridus:3.994735):0.354,Amaranthus_blitoides:4.348735):0.118536,Amaranthus_tricolor:4.467271):0.044084,Amaranthus_tuberculatus:4.511355):3.971122,Amaranthus_albus:8.482477):19.674359,Celosia_argentea:28.156836):15.50449):26.451306):17.737972):13.022313,Simmondsia_chinensis:100.872917):6.178391,(((((Polygonum_aviculare:14.562243,(Fallopia_dumetorum:4.803675,Fallopia_convolvulus:4.803675):9.758568):19.244547,(((Persicaria_longiseta:2.519192,Reynoutria_japonica:2.519192):4.890337,Persicaria_maculosa:7.409529):25.894494,(((Fagopyrum_statice:4.679937,Fagopyrum_lineare:4.679937):13.170878,Fagopyrum_tataricum:17.850815):10.875031,((Fagopyrum_cymosum:8.301119,Fagopyrum_esculentum:8.301119):17.150759,(Fagopyrum_gracilipes:5.747441,Fagopyrum_leptopodum:5.747441):19.704437):3.273968):4.578177):0.502767):8.287391,Coccoloba_uvifera:42.094181):25.807955,Armeria_maritima:67.902136):31.3934,(Drosera_rotundifolia:52.197979,Drosera_peltata:52.197979):47.097557):7.755772)Caryophyllales.rn.d8s.tre:12.822926)mrcaott248ott557:1.510431,((Macrosolen_cochinchinensis:81.472736,(Viscum_album:76.946153,Santalum_album:76.946153):4.526583):15.10183,Ximenia_americana:96.574566):24.810099)mrcaott248ott19688:1.196363,Dillenia_indica:122.581028)mrcaott248ott10053:1.153209,((((((((((((((((((((((Trifolium_fragiferum:3.822032,Trifolium_hybridum:3.822032):0.530766,Trifolium_alexandrinum:4.352798):1.576812,Trifolium_repens:5.92961):2.363513,((Trifolium_medium:2.762471,Trifolium_pratense:2.762471):1.419496,Trifolium_incarnatum:4.181967):4.111156):4.458682,((Trifolium_campestre:0.183433,Trifolium_dubium:0.183433):6.458255,Trifolium_aureum:6.641688):6.110117):4.821848,(((((((Medicago_falcata:0.304663,Medicago_sativa:0.304663):1.623027,Medicago_arborea:1.92769):3.675264,Medicago_polymorpha:5.602954):4.146252,Medicago_arabica:9.749206):0.362389,(Medicago_lupulina:4.332902,Medicago_minima:4.332902):5.778693):4.660104,(Melilotus_officinalis:0.917975,Melilotus_albus:0.917975):13.853724):1.715882,Ononis_spinosa:16.487581):1.086072):0.985589,((((((((Vicia_sativa:3.398378,Vicia_sepium:3.398378):1.599739,Vicia_lathyroides:4.998117):0.243155,Vicia_faba:5.241272):1.734651,Vicia_pannonica:6.975923):2.004433,((Vicia_unijuga:1.057638,(Vicia_ramuliflora:1.053391,Vicia_amurensis:1.053391):0.004247):0.325161,Vicia_amoena:1.382799):7.597557):1.81156,(((Vicia_cracca:0.565313,Vicia_tenuifolia:0.565313):3.472754,Vicia_villosa:4.038067):6.751196,Vicia_hirsuta:10.789263):0.002653):0.553,Vicia_tetrasperma:11.344916):0.015393,((Lathyrus_pratensis:7.003037,Lathyrus_aphaca:7.003037):2.165214,((Lathyrus_tuberosus:5.482946,Lathyrus_latifolius:5.482946):2.016045,Lathyrus_sativus:7.498991):1.66926):2.192058):7.198933):5.815209,Galega_officinalis:24.374451):1.388288,(Cicer_songaricum:8.788419,Cicer_arietinum:8.788419):16.97432):6.49712,((Colutea_arborescens:28.654284,Onobrychis_viciifolia:28.654284):0.376782,(Caragana_sinica:6.564299,Caragana_arborescens:6.564299):22.466767):3.228793):17.166662,((((Lotus_tenuis:1.036986,Lotus_corniculatus:1.036986):4.723709,Lotus_angustissimus:5.760695):17.490825,Coronilla_varia:23.25152):23.006909,(Olneya_tesota:10.193517,Robinia_pseudoacacia:10.193517):36.064912):3.168092):8.204971,((((((((Glycine_tabacina:5.82315,(Glycine_tomentella:2.460354,Glycine_clandestina:2.460354):3.362796):2.690568,Glycine_max:8.513718):21.933361,(((Phaseolus_lunatus:9.739238,(Phaseolus_coccineus:3.789987,Phaseolus_vulgaris:3.789987):5.949251):3.996246,(((((Vigna_radiata:1.991339,Vigna_aconitifolia:1.991339):0.346125,Vigna_trilobata:2.337464):1.518732,Vigna_angularis:3.856196):3.669939,(Vigna_vexillata:4.663543,Vigna_unguiculata:4.663543):2.862592):2.184666,Vigna_luteola:9.710801):4.024683):16.408498,(((Erythrina_vespertilio:2.548746,Erythrina_lysistemon:2.548746):0.004468,Erythrina_humeana:2.553214):13.959603,Psophocarpus_tetragonolobus:16.512817):13.631165):0.303097):1.202173,(Cajanus_cajan:15.480425,(Flemingia_macrophylla:4.048759,Flemingia_strobilifera:4.048759):11.431666):16.168827):0.335024,Butea_monosperma:31.984276):12.069353,(Pongamia_pinnata:17.631466,(Tephrosia_candida:4.556349,Tephrosia_purpurea:4.556349):13.075117):26.422163):0.878918,Clitoria_ternatea:44.932547):5.585023,Cyamopsis_tetragonoloba:50.51757):7.113922):6.584434,((((Crotalaria_lanceolata:9.204984,(Crotalaria_retusa:4.088573,((Crotalaria_verrucosa:2.347581,Crotalaria_juncea:2.347581):0.325029,Crotalaria_sericea:2.67261):1.415963):5.116411):0.085096,(Crotalaria_pallida:8.085408,Crotalaria_incana:8.085408):1.204672):30.696118,(((Lupinus_polyphyllus:9.589447,(Lupinus_luteus:8.364552,Lupinus_angustifolius:8.364552):1.224895):0.948009,Lupinus_albus:10.537456):8.487756,(((Genista_tinctoria:13.8192,Ulex_europaeus:13.8192):1.228452,Cytisus_nigricans:15.047652):0.444616,Cytisus_scoparius:15.492268):3.532944):20.960986):19.221012,((Dalbergia_sissoo:3.995504,Dalbergia_volubilis:3.995504):55.204852,Castanospermum_australe:59.200356):0.006854):5.008716):13.747261,(((((((((((((((((Acacia_concurrens:2.110806,Acacia_mangium:2.110806):2.409204,Acacia_aulacocarpa:4.52001):0.624067,Acacia_longissima:5.144077):0.365944,(Acacia_acuminata:5.044716,Acacia_auriculiformis:5.044716):0.465305):0.791158,((Acacia_rigens:4.344333,Acacia_colletioides:4.344333):0.423916,(Acacia_floribunda:3.067326,Acacia_maidenii:3.067326):1.700923):1.53293):1.142976,Acacia_crassicarpa:7.444155):4.690384,(((((((Acacia_mearnsii:2.769863,Acacia_dealbata:2.769863):0.579206,Acacia_baileyana:3.349069):1.55461,(Acacia_podalyriifolia:4.695388,Acacia_decurrens:4.695388):0.208291):1.255538,Acacia_penninervis:6.159217):0.936706,(Acacia_falcata:3.931342,Acacia_beckleri:3.931342):3.164581):1.497795,Acacia_calamifolia:8.593718):0.449991,Acacia_macradenia:9.043709):3.09083):7.445632,Acacia_acinacea:19.580171):1.675127,Acacia_ampliceps:21.255298):7.370226,(Prosopis_juliflora:16.549171,Prosopis_chilensis:16.549171):12.076353):0.189922,Leucaena_leucocephala:28.815446):0.002853,Leucaena_salvadorensis:28.818299):6.93212,(Adenanthera_microsperma:0.013751,Adenanthera_pavonina:0.013751):35.736668):9.99728,(Parkinsonia_aculeata:28.840053,Peltophorum_pterocarpum:28.840053):16.907646):2.079759,Caesalpinia_pulcherrima:47.827458):2.87239,Cassia_fistula:50.699848):7.403068,(Gleditsia_sinensis:34.947181,Ceratonia_siliqua:34.947181):23.155735):19.860271):6.063258,(Bauhinia_tomentosa:10.989923,(Bauhinia_purpurea:2.797717,Bauhinia_galpinii:2.797717):8.192206):73.036522):0.736893,Tamarindus_indica:84.763338):27.937858,((((((((Potentilla_recta:78.681759,(Comarum_palustre:60.259498,(Fragaria_vesca:7.250576,Fragaria_viridis:7.250576):53.008922):18.422261):2.674252,Agrimonia_eupatoria:81.356011):0.679169,((((((Rosa_canina:34.767572,Rosa_gallica:34.767572):27.298838,Rosa_multiflora:62.06641):2.121317,(Rosa_rugosa:11.085564,(Rosa_willmottiae:4.786004,Rosa_bella:4.786004):6.29956):53.102163):0.15223,(((((((Rosa_moyesii:45.25865,(Rosa_spinosissima:21.686087,Rosa_acicularis:21.686087):23.572563):1.85634,Rosa_foetida:47.11499):0.867975,Rosa_roxburghii:47.982965):0.09493,Rosa_laevigata:48.077895):0.984859,Rosa_banksiae:49.062754):2.321504,(Rosa_sericea:39.597642,Rosa_xanthina:39.597642):11.786616):4.206548,Rosa_chinensis:55.590806):8.749151):6.368331,Rosa_persica:70.708288):0.039947,Rosa_bracteata:70.748235):11.286945):2.967608,((((Rubus_alceifolius:54.820727,Rubus_occidentalis:54.820727):5.227164,(Rubus_crataegifolius:51.006172,Rubus_ellipticus:51.006172):9.041719):0.277926,(((Rubus_parvifolius:28.09947,Rubus_innominatus:28.09947):7.500187,(Rubus_niveus:35.026813,Rubus_lasiostylus:35.026813):0.572844):17.772797,Rubus_idaeus:53.372454):6.953363):10.074409,Rubus_chamaemorus:70.400226):14.602562):8.266487,Dryas_octopetala:93.269275):1.291905,((((((((((((Malus_hupehensis:1.571365,Malus_halliana:1.571365):1.182202,(Malus_yunnanensis:0.53128,Malus_kansuensis:0.53128):2.222287):0.99107,Malus_rockii:3.744637):0.11993,((((Malus_prunifolia:0.136472,Malus_baccata:0.136472):0.492234,Malus_spectabilis:0.628706):0.074462,(Malus_prattii:0.098183,Malus_toringoides:0.098183):0.604985):0.270705,Malus_sikkimensis:0.973873):2.890694):0.013712,Malus_toringo:3.878279):3.865317,(Aronia_arbutifolia:1.138954,Aronia_melanocarpa:1.138954):6.604642):0.674235,Cydonia_oblonga:8.417831):1.196968,(Crataegus_wilsonii:4.175793,Crataegus_pinnatifida:4.175793):5.439006):1.130995,(((Cotoneaster_divaricatus:1.944865,Cotoneaster_dammeri:1.944865):1.333494,Cotoneaster_horizontalis:3.278359):6.635294,(Pyrus_communis:1.74585,Pyrus_calleryana:1.74585):8.167803):0.832141):38.860409,Spiraea_chamaedryfolia:49.606203):1.127065,((((((Prunus_spinosa:8.862027,Prunus_domestica:8.862027):1.405797,(Prunus_armeniaca:9.376205,Prunus_tomentosa:9.376205):0.891619):2.190719,((Prunus_persica:6.302884,(Prunus_kansuensis:1.140871,Prunus_mira:1.140871):5.162013):0.237432,Prunus_davidiana:6.540316):5.918227):5.619952,((Prunus_avium:11.123271,Prunus_cerasus:11.123271):3.738807,Prunus_mahaleb:14.862078):3.216417):8.451809,(Prunus_laurocerasus:21.733365,Prunus_virginiana:21.733365):4.796939):0.399346,Prunus_padus:26.92965):23.803618):4.253191,(Prinsepia_uniflora:51.112883,Rhodotypos_scandens:51.112883):3.873576):39.574721):4.400616,(((((((((Ficus_pumila:8.211575,Ficus_religiosa:8.211575):1.047727,Ficus_carica:9.259302):0.842523,(Ficus_racemosa:6.135127,Ficus_pandurata:6.135127):3.966698):0.761219,((Ficus_benjamina:3.19994,Ficus_elastica:3.19994):2.226812,Ficus_rumphii:5.426752):5.436292):31.600853,Morus_alba:42.463897):26.048553,(Pilea_pumila:42.20782,((Urtica_fissa:13.641878,Urtica_dioica:13.641878):0.628763,Urtica_urens:14.270641):27.937179):26.30463):4.956685,((Humulus_lupulus:16.144623,Cannabis_sativa:16.144623):51.025174,Celtis_bungeana:67.169797):6.299338):5.701507,(((Ulmus_minor:0.536514,Ulmus_glabra:0.536514):3.843763,Ulmus_rubra:4.380277):2.055773,Ulmus_americana:6.43605):72.734592):6.322774,((Colubrina_asiatica:51.398492,((Rhamnus_cathartica:0.780555,Rhamnus_davurica:0.780555):10.176834,Frangula_alnus:10.957389):40.441103):24.21016,(Elaeagnus_umbellata:10.517554,Elaeagnus_angustifolia:10.517554):65.091098):9.884764):13.46838)Rosales.rn.d8s.tre:12.186212,((((((((((Cucumis_sativus:8.410665,Cucumis_melo:8.410665):4.866322,Coccinia_grandis:13.276987):0.34841,(Benincasa_hispida:10.690218,Lagenaria_siceraria:10.690218):2.935179):6.512963,(((Cucurbita_moschata:3.910652,Cucurbita_pepo:3.910652):0.702654,Cucurbita_maxima:4.613306):1.232602,Cucurbita_foetidissima:5.845908):14.292452):3.3158,Echinocystis_lobata:23.45416):0.146766,Ecballium_elaterium:23.600926):3.197818,Momordica_charantia:26.798744):8.726804,Gynostemma_pentaphyllum:35.525548):26.401344,((Begonia_grandis:19.42135,Begonia_cucullata:19.42135):0.811864,(Begonia_coccinea:2.441972,Begonia_maculata:2.441972):17.791242):41.693678):47.141593,((((((((((((Betula_ermanii:21.478677,Betula_pendula:21.478677):3.698854,Betula_costata:25.177531):0.770594,Betula_alnoides:25.948125):2.997465,Betula_schmidtii:28.94559):0.835946,Betula_nana:29.781536):3.602442,Betula_utilis:33.383978):2.492292,Betula_humilis:35.87627):3.771628,Betula_luminifera:39.647898):28.627551,Alnus_glutinosa:68.275449):16.497017,(Casuarina_glauca:4.610205,Casuarina_equisetifolia:4.610205):80.162261):5.876564,Juglans_regia:90.64903):7.230533,(((((Quercus_griffithii:8.67702,(Quercus_variabilis:1.087094,Quercus_acutissima:1.087094):7.589926):2.38702,Quercus_glauca:11.06404):0.712659,Quercus_ellipsoidalis:11.776699):0.532457,((((((Castanopsis_indica:2.632415,Castanopsis_mekongensis:2.632415):0.137059,Castanopsis_rockii:2.769474):0.073856,Castanopsis_echinocarpa:2.84333):0.239366,(Castanopsis_fissa:0.238816,Castanopsis_calathiformis:0.238816):2.84388):0.079349,(((Castanopsis_wattii:0.062894,Castanopsis_delavayi:0.062894):1.381395,Castanopsis_orthacantha:1.444289):0.047205,Castanopsis_fleuryi:1.491494):1.670551):6.815099,((((((Lithocarpus_fohaiensis:2.633961,Lithocarpus_truncatus:2.633961):0.200665,Lithocarpus_fenestratus:2.834626):0.99831,((Lithocarpus_dealbatus:1.236903,Lithocarpus_xylocarpus:1.236903):0.167823,Lithocarpus_echinophorus:1.404726):2.42821):0.058365,Lithocarpus_pachylepis:3.891301):1.631977,(Lithocarpus_hancei:3.203055,Lithocarpus_litseifolius:3.203055):2.320223):0.882929,Lithocarpus_bacgiangensis:6.406207):3.570937):2.332012):0.170691,Trigonobalanus_doichangensis:12.479847):85.399716):11.188922)mrcaott2511ott32687:2.079523)mrcaott371ott2511:1.553188)mrcaott371ott579:3.084369,((((((Hypericum_hirsutum:4.43788,Hypericum_perforatum:4.43788):90.427399,(((((Passiflora_suberosa:13.258908,Passiflora_morifolia:13.258908):8.18759,((((Passiflora_quadrangularis:3.271299,Passiflora_laurifolia:3.271299):0.734127,Passiflora_edulis:4.005426):4.94226,Passiflora_caerulea:8.947686):2.630611,Passiflora_foetida:11.578297):9.868201):25.953211,Turnera_ulmifolia:47.399709):43.726725,(((((Salix_viminalis:0.068194,Salix_caprea:0.068194):0.226413,Salix_cinerea:0.294607):5.87598,Salix_triandra:6.170587):1.171845,(Salix_alba:2.541855,(Salix_babylonica:2.53536,Salix_fragilis:2.53536):0.006495):4.800577):30.69623,((Populus_alba:1.574183,Populus_tremula:1.574183):1.537515,Populus_nigra:3.111698):34.926964):53.087772):3.235621,((Viola_rupestris:3.772946,Viola_hirta:3.772946):3.636747,Viola_tricolor:7.409693):86.952362):0.503224):0.046307,(Bridelia_retusa:71.92514,Bischofia_javanica:71.92514):22.986446):7.696173,(((((((Euphorbia_hirta:10.494044,Euphorbia_pulcherrima:10.494044):9.834491,(Euphorbia_obesa:1.622385003,Euphorbia_pentagona:1.622385003):18.70615):1.579041,(Euphorbia_peplus:17.692817,(Euphorbia_helioscopia:17.130482,Euphorbia_phymatosperma:17.130482):0.562335):4.214759):10.024876,Hura_crepitans:31.932452):19.938402,((Jatropha_curcas:9.257519003,Jatropha_podagrica:9.257519003):37.801751,(Manihot_esculenta:14.149102,Hevea_brasiliensis:14.149102):32.910168):4.811584):10.025653,Ricinus_communis:61.896507):32.31013,(Linum_usitatissimum:92.849234,Ochna_serrulata:92.849234):1.357403):8.401122):8.818668,Averrhoa_carambola:111.426427)mrcaott2ott345:0.594159,(((Euonymus_hamiltonianus:8.47241,((Euonymus_alatus:3.539367,Euonymus_phellomanus:3.539367):3.927627,Euonymus_verrucosus:7.466994):1.005416):16.325969,(Celastrus_scandens:3.301217,Celastrus_orbiculatus:3.301217):21.497162):43.741175,Parnassia_palustris:68.539554):43.481032)mrcaott2ott1479:3.764979)mrcaott2ott371:1.025329,Larrea_tridentata:116.810894)mrcaott2ott2737:1.76771,((((((((((((((((((Sisymbrium_officinale:9.552839,Sisymbrium_irio:9.552839):2.212236,(Sisymbrium_loeselii:9.981266,Sisymbrium_altissimum:9.981266):1.783809):13.221587,((((Brassica_oleracea:4.930144,((Brassica_rapa:3.532235,Brassica_juncea:3.532235):0.095039,Brassica_napus:3.627274):1.30287):18.159137,Sinapis_alba:23.089281):0.066119,Cakile_edentula:23.1554):1.66379,Orychophragmus_violaceus:24.81919):0.167472):0.536633,(Isatis_tinctoria:20.007961,Conringia_planisiliqua:20.007961):5.515334):0.958278,(Alliaria_petiolata:23.821285,Thlaspi_arvense:23.821285):2.660288):0.264212,Cochlearia_officinalis:26.745785):0.188645,((((Draba_fladnizensis:2.981737,(Draba_glomerata:1.852498,Draba_altaica:1.852498):1.129239):3.420028,Draba_nemorosa:6.401765):12.421023,(Arabis_hirsuta:18.18557,Arabis_alpina:18.18557):0.637218):6.394325,Ptilotrichum_canescens:25.217113):1.717317):0.132548,Berteroa_incana:27.066978):0.235294,(((Euclidium_syriacum:11.0433,Christolea_crassifolia:11.0433):10.629186,(Matthiola_incana:20.018884,(Hesperis_matronalis:18.700603,Bunias_orientalis:18.700603):1.318281):1.653602):3.382134,(Parrya_nudicaulis:14.88936,Chorispora_tenella:14.88936):10.16526):2.247652):0.146893,(((((((Erysimum_cheiranthoides:1.605007,Erysimum_diffusum:1.605007):12.327788,(Olimarabidopsis_cabulica:0.8020319997,Olimarabidopsis_pumila:0.8020319997):13.130763):0.641005,(((Arabidopsis_lyrata:8.995538,Arabidopsis_thaliana:8.995538):4.123422,(Capsella_bursa-pastoris:10.744807,Neslia_paniculata:10.744807):2.374153):0.542767,Turritis_glabra:13.661727):0.912073):2.242888,((Crucihimalaya_mollissima:1.500887,Crucihimalaya_wallichii:1.500887):0.140856,Crucihimalaya_himalaica:1.641743):15.174945):6.198952,(((((Cardamine_pratensis:3.845137,Cardamine_flexuosa:3.845137):6.106985,Cardamine_hirsuta:9.952122):1.114345,Cardamine_impatiens:11.066467):4.110201,Nasturtium_officinale:15.176668):1.608522,(Rorippa_palustris:0.0400329997,Rorippa_sylvestris:0.0400329997):16.745157):6.23045):0.423589,((Lepidium_virginicum:4.967589,(Lepidium_sativum:2.896812,Lepidium_densiflorum:2.896812):2.070777):0.274794,Lepidium_apetalum:5.242383):18.196846):0.183,(Hornungia_procumbens:20.443642,Hedinia_tibetica:20.443642):3.178587):3.826936):0.604057,Malcolmia_africana:28.053222):31.422482,Reseda_lutea:59.475704):19.513493,Carica_papaya:78.989197):2.611864,Tropaeolum_majus:81.601061)Brassicales.rn.d8s.tre:11.352076,((((((((Malva_parviflora:0.639873,Malva_sylvestris:0.639873):14.614272,Abutilon_theophrasti:15.254145):3.905965,(((Gossypium_anomalum:5.085068,(Gossypium_herbaceum:0.900433,Gossypium_arboreum:0.900433):4.184635):2.121804,(Gossypium_barbadense:1.07716,Gossypium_hirsutum:1.07716):6.129712):5.472768,Thespesia_populnea:12.67964):6.48047):0.392969,(Hibiscus_cannabinus:12.946556,Abelmoschus_esculentus:12.946556):6.606523):3.145509,((Ceiba_pentandra:10.905378,Bombax_ceiba:10.905378):3.988345,Adansonia_digitata:14.893723):7.804865):2.845133,Pterospermum_lanceifolium:25.543721):12.817413,(Theobroma_cacao:32.280668,((Corchorus_olitorius:4.532938,Corchorus_capsularis:4.532938):4.255687,Corchorus_aestuans:8.788625):23.492043):6.080466):28.431909,(Cistus_creticus:57.746202,Bixa_orellana:57.746202):9.046841):26.160094)mrcaott378ott1697:11.304922,(((((((Phellodendron_amurense:46.161758,(Skimmia_japonica:31.729573,Casimiroa_edulis:31.729573):14.432185):2.847994,Ptelea_trifoliata:49.009752):1.924174,Orixa_japonica:50.933926):3.44479,(((Citrus_medica:21.469917,Aegle_marmelos:21.469917):5.317287,Murraya_paniculata:26.787204):22.876728,(Boenninghausenia_albiflora:36.561308,Ruta_graveolens:36.561308):13.102624):4.714784):14.90101,((Melia_azedarach:50.633933,Khaya_senegalensis:50.633933):16.255865,Ailanthus_altissima:66.889798):2.389928):9.868676,(((((Acer_negundo:11.749799,(Acer_campestre:3.268696,Acer_platanoides:3.268696):8.481103):0.716653,((Acer_monspessulanum:7.252709,Acer_saccharum:7.252709):4.943953,Acer_tataricum:12.196662):0.26979):0.864807,(Acer_saccharinum:1.342015,Acer_rubrum:1.342015):11.989244):31.805872,(Aesculus_chinensis:7.47883,Aesculus_hippocastanum:7.47883):37.658301):12.444139,(Litchi_chinensis:52.721004,Dodonaea_viscosa:52.721004):4.860266):21.567132):0.769939,(Protium_serratum:51.559174,(((Cotinus_coggygria:19.480202,Toxicodendron_radicans:19.480202):5.551777,Mangifera_indica:25.031979):18.321453,Lannea_coromandelica:43.353432):8.205742):28.359167):24.339718)mrcaott96ott378:8.559102,(Staphylea_bumalda:31.286724,Stachyurus_praecox:31.286724):81.530437)mrcaott96ott14140:4.176035,((((((((Eucalyptus_occidentalis:28.399544,Eucalyptus_cornuta:28.399544):2.38108,((Eucalyptus_globulus:21.674998,Eucalyptus_pulverulenta:21.674998):6.073325,(Eucalyptus_camaldulensis:24.303062,((Eucalyptus_grandis:1.176651,Eucalyptus_pellita:1.176651):11.342797,Eucalyptus_saligna:12.519448):11.783614):3.445261):3.032301):1.757918,Eucalyptus_elata:32.538542):3.834945,((Eugenia_uniflora:28.182863,Psidium_guajava:28.182863):7.692667,Syzygium_cumini:35.87553):0.497957):3.859796,(Melaleuca_quinquenervia:1.928333,Melaleuca_leucadendra:1.928333):38.30495):53.086235,(((((Oenothera_biennis:2.367186,Oenothera_laciniata:2.367186):15.716315,Oenothera_rosea:18.083501):7.669345,(((Epilobium_tetragonum:2.899684,Epilobium_palustre:2.899684):0.726114,Epilobium_hirsutum:3.625798):5.522154,Epilobium_ciliatum:9.147952):16.604894):16.31831,Circaea_lutetiana:42.071156):30.564721,((Lagerstroemia_tomentosa:39.115177,Lawsonia_inermis:39.115177):8.691747,(Woodfordia_fruticosa:43.339511,Punica_granatum:43.339511):4.467413):24.828953):20.683641):3.319806,((Terminalia_muelleri:22.832486,Terminalia_bellirica:22.832486):3.519541,(Terminalia_chebula:22.680399,(Terminalia_arjuna:16.125914,Terminalia_myriocarpa:16.125914):6.554485):3.671628):70.287297)Myrtales.rn.d8s.tre:13.050392,Erodium_cicutarium:109.689716)mrcaott607ott1276:7.30348)mrcaott96ott607:1.585408)mrcaott2ott96:2.70865,((((Vitis_betulifolia:20.002455,Vitis_flexuosa:20.002455):10.628659,(Vitis_coignetiae:20.206963,Vitis_amurensis:20.206963):10.424151):4.304708,Vitis_vinifera:34.935822):14.894791,Parthenocissus_tricuspidata:49.830613):71.456641)mrcaott2ott8384:1.117838,((((Graptopetalum_macdougallii:31.1605626,Aeonium_sedifolium:31.1605626):62.4159584,Myriophyllum_spicatum:93.576521):11.106525,(Saxifraga_oppositifolia:84.0779035,Ribes_rubrum:84.0779035):20.6051425):3.216446,(((Distylium_myricoides:82.9779208,Corylopsis_sinensis:82.9779208):11.1431438,(Daphniphyllum_pentandrum:60.493731,Cercidiphyllum_japonicum:60.493731):33.6273336):10.71734,((Paeonia_rockii:2.87460498,Paeonia_delavayi:2.87460498):3.87637702,Paeonia_anomala:6.750982):98.0874226):3.0610874):14.5056)mrcaott2ott2464:1.329145)Pentapetalae:1.341336,Gunnera_manicata:125.075573)Gunneridae:3.478354,((Buxus_microphylla:9.812122,(Buxus_henryi:6.210826,Buxus_harlandii:6.210826):3.601296):50.871424,Pachysandra_terminalis:60.683546):67.870381)mrcaott2ott8379:0.443347,Trochodendron_aralioides:128.997274)mrcaott2ott62529:1.327257,((((Banksia_ericifolia:31.186313,Grevillea_robusta:31.186313):11.262993,Protea_cynaroides:42.449306):63.772327,Platanus_orientalis:106.221633):21.278961,Nelumbo_nucifera:127.500594)Proteales.rn.d8s.tre:2.823937)mrcaott2ott969:1.356169,(((((((((((((((Ranunculus_polyanthemos:5.090957,Ranunculus_muricatus:5.090957):0.66064,Ranunculus_repens:5.751597):1.237417,Ranunculus_acris:6.989014):0.248001,Ranunculus_arvensis:7.237015):1.568145,(Ranunculus_reptans:0.676871,Ranunculus_lingua:0.676871):8.128289):4.473828,(Ranunculus_gmelinii:9.798883,((Ranunculus_trichophyllus:3.360928,Ranunculus_circinatus:3.360928):1.97473,Ranunculus_sceleratus:5.335658):4.463225):3.480105):25.539868,(Clematis_montana:19.232421,((Anemone_coronaria:12.034304,(Hepatica_nobilis:3.301328,Hepatica_henryi:3.301328):8.732976):1.077435,Pulsatilla_patens:13.111739):6.120682):19.586435):1.725596,Helleborus_thibetanus:40.544452):0.747163,Caltha_palustris:41.291615):0.494432,(Delphinium_ajacis:40.712192,Nigella_damascena:40.712192):1.073855):0.764671,(((((((Thalictrum_alpinum:2.98665,Thalictrum_virgatum:2.98665):0.470881,Thalictrum_diffusiflorum:3.457531):0.2299,Thalictrum_isopyroides:3.687431):0.118348,Thalictrum_omeiense:3.805779):0.222613,Thalictrum_aquilegiifolium:4.028392):0.552203,(((Thalictrum_simplex:0.836086,Thalictrum_flavum:0.836086):0.66927,Thalictrum_minus:1.505356):1.471366,(Thalictrum_delavayi:1.649692,Thalictrum_grandiflorum:1.649692):1.32703):1.603873):3.805235,((Thalictrum_filamentosum:2.208828,Thalictrum_rubescens:2.208828):1.168849,(Thalictrum_clavatum:2.736819,Thalictrum_ichangense:2.736819):0.640858):5.008153):34.164888):37.732339,((((Berberis_thunbergii:0.733418,Berberis_koreana:0.733418):1.055788,Berberis_soulieana:1.789206):0.292375,Berberis_gilgiana:2.081581):40.860576,Nandina_domestica:42.942157):37.3409):17.915206,Akebia_trifoliata:98.198263):14.739821,((((Papaver_nudicaule:21.115282,((Papaver_orientale:5.534347,Papaver_somniferum:5.534347):1.273623,(Papaver_dubium:5.314466,Papaver_rhoeas:5.314466):1.493504):14.307312):34.029506,(Chelidonium_majus:45.705218,Sanguinaria_canadensis:45.705218):9.43957):9.823151,Eschscholzia_californica:64.967939):22.23009,Dicentra_canadensis:87.198029):25.740055):1.894891,Euptelea_pleiosperma:114.832975)Ranunculales.rn.d8s.tre:16.847725)eudicotyledons:2.742485,Ceratophyllum_demersum:134.423185)mrcaott2ott10930:1.33488,((((((((((((((((Cymbidium_sinense:26.437425,((((((Vanda_lamellata:4.054316,Vanda_cristata:4.054316):8.429084,Rhynchostylis_retusa:12.4834):0.789701,(((Schoenorchis_gemmata:9.517399,Cleisostoma_racemiferum:9.517399):0.907665,Gastrochilus_calceolaris:10.425064):0.003432,Smitinandia_micrantha:10.428496):2.844605):0.005133,Aerides_odorata:13.278234):3.600019,(((((Phalaenopsis_amabilis:4.006283,Phalaenopsis_aphrodite:4.006283):6.497805,Phalaenopsis_equestris:10.504088):0.998084,Phalaenopsis_wilsonii:11.502172):1.256634,Phalaenopsis_deliciosa:12.758806):0.891214,Phalaenopsis_mannii:13.65002):3.228233):7.308988,Polystachya_concreta:24.187241):2.250184):4.005023,Corallorhiza_trifida:30.442448):0.734044,(Calanthe_tricarinata:11.899843,Phaius_wallichii:11.899843):19.276649):1.055384,(((((Dendrobium_crumenatum:15.578949,Dendrobium_densiflorum:15.578949):0.44212,(Dendrobium_wattii:3.588202,Dendrobium_bellatulum:3.588202):12.432867):0.552265,((Dendrobium_parishii:6.830256,Dendrobium_aphyllum:6.830256):3.464768,(Dendrobium_fimbriatum:8.907461,(Dendrobium_moschatum:5.936187,Dendrobium_pulchellum:5.936187):2.971274):1.387563):6.27831):1.290392,Dendrobium_lindleyi:17.863726):8.309931,(Bulbophyllum_medusae:14.075597,Bulbophyllum_reptans:14.075597):12.09806):6.058219):0.947408,((Coelogyne_fimbriata:3.713728,Coelogyne_flaccida:3.713728):7.512582,Bletilla_striata:11.22631):21.952974):10.857544,(Epipactis_helleborine:23.254723,(Cephalanthera_damasonium:2.909046,Cephalanthera_longifolia:2.909046):20.345677):20.782105):9.85901,((((Platanthera_bifolia:8.73057,Platanthera_chlorantha:8.73057):0.758671,Gymnadenia_conopsea:9.489241):12.443953,Habenaria_rhodocheila:21.933194):24.14935,(Goodyera_repens:23.177028,Ludisia_discolor:23.177028):22.905516):7.813294):5.849482,Vanilla_planifolia:59.74532):0.001585,(((((Paphiopedilum_purpuratum:4.465193,(Paphiopedilum_wardii:3.175539,Paphiopedilum_appletonianum:3.175539):1.289654):3.756119,((((Paphiopedilum_villosum:1.462696,Paphiopedilum_insigne:1.462696):0.275715,Paphiopedilum_gratrixianum:1.738411):4.355778,Paphiopedilum_exul:6.094189):1.666191,Paphiopedilum_dianthum:7.76038):0.460932):1.896401,Paphiopedilum_concolor:10.117713):2.257286,(Paphiopedilum_micranthum:5.837052,Paphiopedilum_armeniacum:5.837052):6.537947):19.674604,((((Cypripedium_calceolus:3.741094,Cypripedium_henryi:3.741094):0.824266,Cypripedium_macranthos:4.56536):9.313796,(Cypripedium_japonicum:2.617016,Cypripedium_formosanum:2.617016):11.26214):1.89273,Cypripedium_flavum:15.771886):16.277717):27.697302):6.73588,(Apostasia_wallichii:3.787189,Apostasia_odorata:3.787189):62.695596):42.475952,((((((((((((Polygonatum_odoratum:4.916786,Polygonatum_verticillatum:4.916786):4.612823,(Maianthemum_japonicum:3.667994,(Maianthemum_canadense:0.004921,Maianthemum_bifolium:0.004921):3.663073):5.861615):0.636795,((Ophiopogon_japonicus:5.055017,(Liriope_muscari:0.744364,Liriope_spicata:0.744364):4.310653):4.648477,(((Aspidistra_elatior:5.487416,(Rohdea_japonica:3.896448,Reineckea_carnea:3.896448):1.590968):0.44264,Convallaria_majalis:5.930056):2.851228,Beaucarnea_recurvata:8.781284):0.92221):0.46291):15.495572,Ruscus_aculeatus:25.661976):14.127822,(Asparagus_densiflorus:2.656659,(Asparagus_officinalis:2.017231,Asparagus_cochinchinensis:2.017231):0.639428):37.133139):2.209968,Cordyline_fruticosa:41.999766):16.67995,((((((Agave_sisalana:0.005871,Agave_americana:0.005871):2.276943,Agave_attenuata:2.282814):13.598527,Yucca_gloriosa:15.881341):1.331509,(Hosta_ventricosa:0.742084,Hosta_plantaginea:0.742084):16.470766):5.061806,Chlorophytum_comosum:22.274656):19.989723,Anemarrhena_asphodeloides:42.264379):16.415337):0.506363,Ledebouria_socialis:59.186079):3.307291,(((((((((((Allium_obliquum:3.789928,Allium_saxatile:3.789928):5.385554,((Allium_splendens:3.107557,Allium_lineare:3.107557):5.826653,Allium_pallasii:8.93421):0.241272):0.778232,(Allium_carolinianum:8.870292,(Allium_macrostemon:6.101787,Allium_caeruleum:6.101787):2.768505):1.083422):0.2068,(Allium_galanthum:5.499329,(Allium_fistulosum:1.954786,Allium_altaicum:1.954786):3.544543):4.661185):0.223273,Allium_chinense:10.383787):0.432638,((Allium_nutans:0.837009,Allium_tenuissimum:0.837009):9.38298,Allium_mongolicum:10.219989):0.596436):0.636165,Allium_mairei:11.45259):1.081814,(Allium_ramosum:0.987601,Allium_tuberosum:0.987601):11.546803):0.413866,Allium_neriniflorum:12.94827):19.059551,Tulbaghia_violacea:32.007821):23.202194,((((((((Narcissus_pseudonarcissus:8.260076,Narcissus_jonquilla:8.260076):2.404765,Narcissus_tazetta:10.664841):2.886608,Leucojum_aestivum:13.551449):0.914667,Lycoris_aurea:14.466116):0.318782,(Zephyranthes_candida:3.936717,Sprekelia_formosissima:3.936717):10.848181):4.801684,(Scadoxus_multiflorus:13.08532,(Clivia_miniata:6.01176,Clivia_nobilis:6.01176):7.07356):6.501262):9.432496,(Crinum_moorei:5.442742,Crinum_asiaticum:5.442742):23.576336):19.123844,Agapanthus_africanus:48.142922):7.067093):7.283355):5.057108,(((Aloe_vera:2.611536,Aloe_marlothii:2.611536):20.623356,Kniphofia_uvaria:23.234892):29.03544,(Hemerocallis_lilioasphodelus:44.117263,Dianella_ensifolia:44.117263):8.153069):15.280146):13.107168,(((Iris_tectorum:18.19609,(Iris_versicolor:6.475674,(Iris_sibirica:4.059381,Iris_pseudacorus:4.059381):2.416293):11.720416):19.873358,Sisyrinchium_rosulatum:38.069448):17.589962,Crocus_sativus:55.65941):24.998236):6.043128,Ixiolirion_tataricum:86.700774):22.257963)Asparagales.rn.d8s.tre:5.641826,((((((((((((((Panicum_virgatum:10.61003,(Panicum_dichotomiflorum:0.903206,Panicum_miliaceum:0.903206):9.706824):3.584198,Eriochloa_villosa:14.194228):0.407303,(Cenchrus_echinatus:6.017531,(Setaria_palmifolia:5.330148,(((Setaria_viridis:1.325415,Setaria_faberi:1.325415):0.171054,Setaria_italica:1.496469):1.261407,Setaria_verticillata:2.757876):2.572272):0.687383):8.584):2.004986,((Echinochloa_crus-galli:0.294809,Echinochloa_frumentacea:0.294809):1.012268,Echinochloa_colonum:1.307077):15.29944):1.297457,(Digitaria_longiflora:6.221507,(Digitaria_ciliaris:1.88748,Digitaria_sanguinalis:1.88748):4.334027):11.682467):2.915135,((((((Sorghum_nitidum:6.548265,((Sorghum_bicolor:0.921664,Sorghum_halepense:0.921664):1.111589,Sorghum_propinquum:2.033253):4.515012):0.141863,((Saccharum_officinarum:2.75196,Saccharum_spontaneum:2.75196):0.15337,(Miscanthus_sinensis:1.295295,Miscanthus_sacchariflorus:1.295295):1.610035):3.784798):0.0051,Imperata_cylindrica:6.695228):1.434482,(Eremochloa_ophiuroides:7.662021,Coix_lacryma-jobi:7.662021):0.467689):6.745387,Zea_mays:14.875097):5.287245,(((Paspalum_dilatatum:0.820381,Paspalum_urvillei:0.820381):2.483826,Paspalum_notatum:3.304207):0.150464,Paspalum_conjugatum:3.454671):16.707671):0.656767):2.50413,(Centotheca_lappacea:22.988852,(Lophatherum_gracile:18.737977,Chasmanthium_latifolium:18.737977):4.250875):0.334387):2.342347,(((((((Cynodon_dactylon:9.070347,Chloris_gayana:9.070347):0.67479,(Eleusine_coracana:1.397099,Eleusine_indica:1.397099):8.348038):2.475721,Dactyloctenium_aegyptium:12.220858):1.196922,((Bouteloua_curtipendula:8.809046,Bouteloua_gracilis:8.809046):4.118627,Tragus_racemosus:12.927673):0.490107):2.218237,Zoysia_japonica:15.636017):1.778343,Eragrostis_minor:17.41436):7.671642,(Isachne_globosa:23.536565,Arundo_donax:23.536565):1.549437):0.579584):14.085492,(((((((((((((Festuca_rupicola:0.237381,Festuca_valesiaca:0.237381):7.184118,(Festuca_altaica:7.398516,Festuca_extremiorientalis:7.398516):0.022983):4.224026,((((Lolium_persicum:0.619552,Lolium_rigidum:0.619552):0.330972,Lolium_multiflorum:0.950524):0.866224,Lolium_temulentum:1.816748):7.247132,Lolium_perenne:9.06388):2.581645):1.018656,((Parapholis_incurva:9.382921,Cynosurus_cristatus:9.382921):2.386745,Dactylis_glomerata:11.769666):0.894515):0.034779,(Aira_caryophyllea:12.550661,Holcus_lanatus:12.550661):0.148299):0.559832,((((Alopecurus_pratensis:3.565846,Alopecurus_myosuroides:3.565846):2.977909,(Phleum_pratense:0.770123,Phleum_alpinum:0.770123):5.773632):1.034211,Milium_effusum:7.577966):4.504359,((Puccinellia_stricta:4.066616,Sclerochloa_dura:4.066616):0.814881,Catabrosa_aquatica:4.881497):7.200828):1.176467):3.634498,((((((((Agrostis_stolonifera:1.771285,Agrostis_vinealis:1.771285):0.00243,Agrostis_canina:1.773715):1.175599,(Agrostis_capillaris:1.188764,Agrostis_gigantea:1.188764):1.76055):1.562402,Calamagrostis_arundinacea:4.511716):8.34841,((Briza_media:3.752559,Briza_minor:3.752559):4.216994,Briza_maxima:7.969553):4.890573):0.052734,Anthoxanthum_odoratum:12.91286):0.244444,(Phalaris_paradoxa:4.729504,Phalaris_arundinacea:4.729504):8.4278):0.004016,((Koeleria_macrantha:7.543708,Trisetum_spicatum:7.543708):1.605547,((((Avena_barbata:0.711074,Avena_sativa:0.711074):1.625383,Avena_fatua:2.336457):1.635847,Avena_eriantha:3.972304):3.899128,Arrhenatherum_elatius:7.871432):1.277823):4.012065):3.73197):2.636326,((((((((Leymus_chinensis:1.285397,(Leymus_secalinus:0.550685,Leymus_racemosus:0.550685):0.734712):3.589531,Psathyrostachys_juncea:4.874928):0.106307,((Hordeum_jubatum:2.206212,(Hordeum_bogdanii:1.273006,Hordeum_brevisubulatum:1.273006):0.933206):0.578281,(Hordeum_vulgare:1.543361,Hordeum_bulbosum:1.543361):1.241132):2.196742):0.194937,(((Agropyron_mongolicum:0.01169,Agropyron_cristatum:0.01169):1.143406,Agropyron_desertorum:1.155096):1.017741,Eremopyrum_triticeum:2.172837):3.003335):0.3073,((((Elymus_dahuricus:1.173418,Elymus_canadensis:1.173418):1.047596,Elymus_abolinii:2.221014):0.279925,Elymus_ciliaris:2.500939):0.327702,((Elymus_sibiricus:1.122511,Elymus_mutabilis:1.122511):0.003908,Elymus_caninus:1.126419):1.702222):2.654831):0.277777,Secale_cereale:5.761249):0.002598,((((Aegilops_umbellulata:0.302664,Aegilops_biuncialis:0.302664):0.387462,Aegilops_triuncialis:0.690126):0.744004,(Aegilops_cylindrica:0.824988,Aegilops_ventricosa:0.824988):0.609142):1.147197,((Triticum_aestivum:0.879015,Triticum_turgidum:0.879015):1.696914,Triticum_monococcum:2.575929):0.005398):3.18252):4.676522,(((((((((Bromus_danthoniae:0.706117,Bromus_squarrosus:0.706117):0.620446,Bromus_japonicus:1.326563):0.382693,Bromus_intermedius:1.709256):0.376744,(Bromus_arvensis:0.903205,Bromus_secalinus:0.903205):1.182795):0.095244,Bromus_scoparius:2.181244):0.808654,(((Bromus_hordeaceus:0.84434,Bromus_commutatus:0.84434):0.079474,Bromus_racemosus:0.923814):0.505025,Bromus_brachystachys:1.428839):1.561059):1.249227,(((Bromus_madritensis:0.333922,Bromus_rubens:0.333922):0.545653,Bromus_tectorum:0.879575):0.904609,(Bromus_sterilis:1.625652,Bromus_diandrus:1.625652):0.158532):2.454941):1.002988,(Bromus_inermis:2.358728,Bromus_erectus:2.358728):2.883385):1.300526,Bromus_carinatus:6.542639):3.89773):9.089247):1.622682,((Brachypodium_sylvaticum:1.723732,Brachypodium_pinnatum:1.723732):3.70597,Brachypodium_distachyon:5.429702):15.722596):2.415178,(Melica_ciliata:15.327321,Glyceria_maxima:15.327321):8.240155):6.218058,Brachyelytrum_erectum:29.785534):4.118054,((((((((Dendrocalamus_brandisii:0.330712,Dendrocalamus_hamiltonii:0.330712):0.003788,Dendrocalamus_asper:0.3345):0.003251,Dendrocalamus_strictus:0.337751):0.006526,Dendrocalamus_giganteus:0.344277):1.998882,((Bambusa_tuldoides:0.530143,Bambusa_multiplex:0.530143):1.81093,((Bambusa_tulda:2.160225,Bambusa_vulgaris:2.160225):0.102235,Bambusa_textilis:2.26246):0.078613):0.002086):0.001922,Thyrsostachys_siamensis:2.345081):1.846504,(Schizostachyum_jaculans:2.279841,Melocanna_baccifera:2.279841):1.911744):19.771377,(Phyllostachys_glauca:1.147377,Phyllostachys_nigra:1.147377):22.815585):9.940626):1.751188,((((Oryza_latifolia:2.435982,Oryza_officinalis:2.435982):4.349546,(Oryza_sativa:1.416023,Oryza_rufipogon:1.416023):5.369505):8.054377,Leersia_oryzoides:14.839905):3.29018,(Zizania_aquatica:12.719357,Hygroryza_aristata:12.719357):5.410728):17.524691):4.096302):34.937353,Flagellaria_indica:74.688431):14.393897,Eriocaulon_aquaticum:89.082328):4.739179,(((((((((((((((Carex_pisiformis:2.820262,Carex_maximowiczii:2.820262):0.710161,(((((Carex_rostrata:1.017505,Carex_lurida:1.017505):0.002599,Carex_vesicaria:1.020104):0.002375,Carex_riparia:1.022479):0.003627,Carex_pseudocyperus:1.026106):1.278873,(Carex_pumila:1.091787,Carex_lasiocarpa:1.091787):1.213192):1.225444):0.003505,((Carex_atrofusca:2.086079,Carex_limosa:2.086079):1.14007,Carex_capillaris:3.226149):0.307779):0.076359,Carex_lanceolata:3.610287):0.340051,((((Carex_buxbaumii:1.440235,(Carex_atrata:0.823579,Carex_parviflora:0.823579):0.616656):2.16189,Carex_sylvatica:3.602125):0.001348,Carex_vaginata:3.603473):0.082241,Carex_acutiformis:3.685714):0.264624):0.388302,Carex_xiphium:4.33864):0.563668,Carex_ussuriensis:4.902308):0.280277,Carex_bostrychostigma:5.182585):0.743969,((((((Carex_lachenalii:0.813384,Carex_tenuiflora:0.813384):0.432279,(Carex_canescens:0.96462,Carex_brunnescens:0.96462):0.281043):0.583996,((Carex_enervis:1.651336,Carex_stipata:1.651336):0.176458,Carex_diandra:1.827794):0.001865):0.18894,Carex_echinata:2.018599):1.083626,(((Carex_kobomugi:1.539608,Carex_disperma:1.539608):0.933451,Carex_leiorhyncha:2.473059):0.207031,Carex_nubigena:2.68009):0.422135):0.726327,Carex_gibba:3.828552):2.098002):0.183314,Carex_siderosticta:6.109868):0.588666,((Carex_obtusata:4.867723,Carex_capillacea:4.867723):0.75016,Carex_microglochin:5.617883):1.080651):8.156116,(Scirpus_sylvaticus:4.102596,(Eriophorum_angustifolium:0.916867,Eriophorum_vaginatum:0.916867):3.185729):10.752054):11.579026,((((Cyperus_haspan:5.146299,Cyperus_rotundus:5.146299):0.085681,Cyperus_iria:5.23198):15.165422,Schoenoplectus_lacustris:20.397402):2.861776,(((Eleocharis_palustris:2.402663,Eleocharis_ovata:2.402663):5.432047,(Eleocharis_uniglumis:3.297181,Eleocharis_acicularis:3.297181):4.537529):2.471171,Eleocharis_quinqueflora:10.305881):12.953297):3.174498):28.727304,((((Juncus_bufonius:7.873451,Juncus_tenuis:7.873451):9.403502,(Juncus_effusus:6.235109,Juncus_inflexus:6.235109):11.041844):15.889722,Juncus_articulatus:33.166675):10.45473,((Luzula_spicata:5.892177,(Luzula_campestris:2.204112,Luzula_sudetica:2.204112):3.688065):6.920672,Luzula_multiflora:12.812849):30.808556):11.539575):22.006993,Mayaca_fluviatilis:77.167973):16.653534):7.154022,((Billbergia_nutans:11.231334,Ananas_comosus:11.231334):64.658547,(Typha_latifolia:10.347611,Typha_angustifolia:10.347611):65.54227):25.085648)Poales.rn.d8s.tre:7.259624,((((((((Zingiber_mioga:4.947016,Zingiber_officinale:4.947016):7.31914,(Curcuma_longa:1.798447,Curcuma_aromatica:1.798447):10.467709):0.463551,Roscoea_purpurea:12.729707):7.180208,Elettaria_cardamomum:19.909915):27.778845,((Maranta_leuconeura:10.421322,Maranta_arundinacea:10.421322):27.817374,Canna_indica:38.238696):9.450064):6.982628,((Strelitzia_reginae:11.371831,Strelitzia_nicolai:11.371831):20.355781,Ravenala_madagascariensis:31.727612):22.943776):25.131837,((Pontederia_cordata:62.333782,Philydrum_lanuginosum:62.333782):6.214232,(((((Tradescantia_virginiana:5.266244,Tradescantia_sillamontana:5.266244):1.49924,Tradescantia_pallida:6.765484):2.850666,Tradescantia_spathacea:9.61615):7.518212,Callisia_repens:17.134362):18.321118,((Commelina_diffusa:4.465464,Commelina_communis:4.465464):1.660511,Commelina_benghalensis:6.125975):29.329505):33.092534)Commelinales.rn.d8s.tre:11.255211)mrcaott121ott3449:18.40578,(((((Areca_concinna:12.48593837,Dypsis_lutescens:12.48593837):8.26436553,(Elaeis_guineensis:15.18241816,((Syagrus_romanzoffiana:7.189896926,Jubaea_chilensis:7.189896926):1.0996355,Cocos_nucifera:8.289532426):6.89288573):5.567885744):23.5821197,((((((Rhapis_excelsa:7.68912752,Guihaia_argyrata:7.68912752):2.6331666,Trachycarpus_nanus:10.32229412):8.04357882,Washingtonia_filifera:18.36587294):12.93502256,((Phoenix_dactylifera:5.6472865,Phoenix_roebelenii:5.6472865):1.183474,Phoenix_canariensis:6.8307605):24.470135):1.9832281,Sabal_minor:33.2841236):4.366583,(((Caryota_mitis:3.57421865,Caryota_urens:3.57421865):27.22233665,(Borassus_flabellifer:18.8322207,Bismarckia_nobilis:18.8322207):11.9643346):1.3637929,Chuniophoenix_nana:32.1603482):5.4903584):6.681717):3.5816274,Nypa_fruticans:47.914051):1.657522,(Calamus_tetradactylus:2.164858855,Calamus_viminalis:2.164858855):47.40671414):48.637432)mrcaott121ott4575:10.026148)mrcaott121ott252:6.36541)mrcaott121ott334:2.276244,(((((((((Lilium_henryi:9.720178,((((Lilium_martagon:4.124771,Lilium_pumilum:4.124771):0.206086,Lilium_davidii:4.330857):1.179342,(Lilium_formosanum:2.070642,Lilium_longiflorum:2.070642):3.439557):2.795519,Lilium_candidum:8.305718):1.41446):3.050138,(((Fritillaria_thunbergii:5.494432,(Fritillaria_verticillata:2.637079,Fritillaria_pallidiflora:2.637079):2.857353):2.452294,Fritillaria_imperialis:7.946726):2.881582,Fritillaria_davidii:10.828308):1.942008):0.051761,Fritillaria_maximowiczii:12.822077):2.651256,Cardiocrinum_giganteum:15.473333):0.534423,Notholirion_bulbuliferum:16.007756):19.49041,(Gagea_lutea:32.854375,(((Tulipa_iliensis:7.532381,(((Tulipa_sylvestris:4.126285,Tulipa_regelii:4.126285):0.378711,Tulipa_dasystemon:4.504996):0.657337,Tulipa_biflora:5.162333):2.370048):1.20298,(Tulipa_uniflora:4.231156,Tulipa_heteropetala:4.231156):4.504205):5.522453,(Amana_edulis:10.647547,Erythronium_japonicum:10.647547):3.610267):18.596561):2.643791):9.527078,Tricyrtis_macropoda:45.025244):10.550942,Smilax_aspera:55.576186):32.331936,((((Colchicum_arenarium:5.521235,Colchicum_autumnale:5.521235):13.144625,Gloriosa_superba:18.66586):14.144113,(Disporum_sessile:1.726433,Disporum_uniflorum:1.726433):31.08354):51.375279,(((Trillium_camschatcense:2.815832,Trillium_tschonoskii:2.815832):8.447839,((((Paris_mairei:4.312271,Paris_polyphylla:4.312271):1.59284,(Paris_forrestii:5.095744,Paris_thibetica:5.095744):0.809367):3.31528,(Paris_quadrifolia:1.458166,Paris_verticillata:1.458166):7.762225):2.022256,Trillium_govanianum:11.242647):0.021024):56.18404,((Veratrum_lobelianum:1.858881,Veratrum_grandiflorum:1.858881):11.681713,((Veratrum_nigrum:2.591637,Veratrum_maackii:2.591637):0.010789,Veratrum_schindleri:2.602426):10.938168):53.907117):16.737541):3.72287):28.968685)mrcaott121ott1439:2.895004,((Pandanus_tectorius:51.467682,Stemona_tuberosa:51.467682):59.010879,((((((Dioscorea_alata:10.40676,Dioscorea_esculenta:10.40676):1.904085,Dioscorea_bulbifera:12.310845):2.274949,Dioscorea_elephantipes:14.585794):14.442366,Dioscorea_zingiberensis:29.02816):32.725907,(Tacca_chantrieri:10.872006,Tacca_plantaginea:10.872006):50.882061):18.150023,Burmannia_disticha:79.90409):30.574471)mrcaott2256ott12550:9.29325)mrcaott121ott2256:11.026948,(((((((((((((Arisaema_ciliatum:78.907348,Arisaema_flavum:78.907348):10.234017,Pinellia_pedatisecta:89.141365):0.037604,Typhonium_trilobatum:89.178969):2.681142,((Alocasia_cucullata:21.511015,Alocasia_longiloba:21.511015):66.113787,(Remusatia_vivipara:50.581368,Colocasia_esculenta:50.581368):37.043434):4.235309):1.218212,Pistia_stratiotes:93.078323):16.037684,Amorphophallus_paeoniifolius:109.116007):1.246582,(Caladium_bicolor:108.117474,Zamioculcas_zamiifolia:108.117474):2.245115):8.231575,(Philodendron_erubescens:118.57676,(Zantedeschia_aethiopica:39.530549,Zantedeschia_albomaculata:39.530549):79.046211):0.017404):0.261387,Calla_palustris:118.855551):2.426305,(Epipremnum_aureum:22.778689,Monstera_deliciosa:22.778689):98.503167):0.368896,(Lemna_minor:73.079776,Spirodela_polyrhiza:73.079776):48.570976):4.569766,Symplocarpus_foetidus:126.220518):2.64086,(((((((Potamogeton_nodosus:4.442511,Potamogeton_crispus:4.442511):26.435614,Zannichellia_palustris:30.878125):22.355932,Zostera_marina:53.234057):15.523485,Triglochin_maritima:68.757542):9.438673,Aponogeton_undulatus:78.196215):1.389981,Scheuchzeria_palustris:79.586196):17.047052,((((Sagittaria_latifolia:1.209335,Sagittaria_sagittifolia:1.209335):37.662004,(Hydrocleys_nymphoides:29.978077,Limnocharis_flava:29.978077):8.893262):1.302791,(Alisma_plantago-aquatica:3.614443,Alisma_lanceolatum:3.614443):36.559687):31.880621,((Najas_marina:44.943836,Hydrocharis_morsus-ranae:44.943836):18.735136,Butomus_umbellatus:63.678972):8.375779):24.578497):32.22813)Alismatales.rn.d8s.tre:1.937381)mrcaott121ott290:2.435645,(Acorus_calamus:30.000014,Acorus_gramineus:30.000014)Acorales.rn.d8s.tre:103.23439)Liliopsida:2.523661)mrcaott2ott121:0.154122,((((((((((Annona_reticulata:9.994698,Annona_squamosa:9.994698):5.420015,Annona_glabra:15.414713):9.961181,Asimina_triloba:25.375894):30.120605,Polyalthia_suberosa:55.496499):0.82121,Cananga_odorata:56.317709):52.062393,Liriodendron_tulipifera:108.380102):1.208338,Myristica_fragrans:109.58844)Magnoliales.rn.d8s.tre:18.112747,(((Cinnamomum_camphora:10.018815,Litsea_glutinosa:10.018815):15.234767,Persea_americana:25.253582):89.648562,(Chimonanthus_praecox:35.095331,Calycanthus_floridus:35.095331):79.806813)Laurales.rn.d8s.tre:12.799043)mrcaott890ott9684:4.692694,((((Peperomia_blanda:8.87284,Peperomia_obtusifolia:8.87284):30.479791,((Piper_nigrum:16.320272,Piper_longum:16.320272):2.238566,Piper_betle:18.558838):20.793793):26.165938,(Saururus_cernuus:44.501597,Houttuynia_cordata:44.501597):21.016972):39.92445,((((Asarum_porphyronotum:0.952105,Asarum_splendens:0.952105):2.018649,Asarum_sieboldii:2.970754):2.009334,Asarum_pulchellum:4.980088):14.837511,Saruma_henryi:19.817599):85.62542)Piperales.rn.d8s.tre:26.950862)Magnoliidae:2.226081,(Chloranthus_spicatus:101.804716,Sarcandra_glabra:101.804716):32.815246)mrcaott890ott34978:1.292225)Mesangiospermae:1.781936,((((Schisandra_sphenanthera:7.487309,Schisandra_rubriflora:7.487309):6.264363,Kadsura_coccinea:13.751672):6.009354,Kadsura_japonica:19.761026):29.930042,((Illicium_anisatum:3.407268,Illicium_henryi:3.407268):2.013854,Illicium_simonsii:5.421122):44.269946):88.003055)mrcaott2ott35778:1.270898,(((((Nymphaea_nouchali:26.128163,Nymphaea_ampla:26.128163):23.30678,((Nymphaea_carpentariae:8.150829,Nymphaea_gigantea:8.150829):7.077845,(Nymphaea_atrans:4.864019,Nymphaea_immutabilis:4.864019):10.364655):34.206269):34.971893,(((Nymphaea_tetragona:25.572276,Nymphaea_mexicana:25.572276):7.037071,(Nymphaea_candida:0.496339,Nymphaea_alba:0.496339):32.113008):33.586433,(Victoria_amazonica:36.813545,Euryale_ferox:36.813545):29.382235):18.211056):4.719494,(Nymphaea_amazonum:65.928267,(Nymphaea_pubescens:19.49692,Nymphaea_lotus:19.49692):46.431347):23.198063):20.448147,((Nuphar_japonica:11.913575,Nuphar_lutea:11.913575):86.49323,Brasenia_schreberi:98.406805):11.167672):29.390544)mrcaott2ott2645;
